# Impact of *Plasmodium falciparum pfhrp2* and *pfhrp3* gene deletions on malaria control worldwide: a systematic review and meta-analysis

**DOI:** 10.1186/s12936-021-03812-0

**Published:** 2021-06-22

**Authors:** Irene Molina-de la Fuente, Andrea Pastor, Zaida Herrador, Agustín Benito, Pedro Berzosa

**Affiliations:** 1 Department of Biomedicine and Biotechnology, School of Pharmacy, University of Alcalá, Alcalá de Henares, Madrid, Spain; 2 Malaria and Neglected Diseases Laboratory, National Centre of Tropical Medicine, Institute of Health Carlos III, 28029 Madrid, Spain; 3 Public Health and Epidemiology Research Group, School of Medicine, University of Alcalá, 28871 Alcalá de Henares, Madrid, Spain; 4 National Centre of Epidemiology, Institute of Health Carlos III, 28029 Madrid, Spain; 5 Network Biomedical Research on Tropical Diseases (RICET in Spanish), Madrid, Spain

**Keywords:** Malaria diagnosis, Rapid diagnostic test, *pfhrp2*, Deletions, Malaria control, RDT

## Abstract

**Background:**

Deletion of *pfhrp2* and/or *pfhrp3* genes cause false negatives in malaria rapid diagnostic test (RDT) and threating malaria control strategies. This systematic review aims to assess the main methodological aspects in the study of *pfhrp2* and *pfhrp3* gene deletions and its global epidemiological status, with special focus on their distribution in Africa; and its possible impact in RDT.

**Methods:**

The systematic review was conducted by examining the principal issues of study design and methodological workflow of studies addressing *pfhrp2* deletion. Meta-analysis was applied to represent reported prevalences of *pfhrp2* and *pfhrp3* single and double deletion in the World Health Organization (WHO) region. Pooled-prevalence of deletions was calculated using DerSimonnian-Laird random effect model. Then, in-deep analysis focused on Africa was performed to assess possible variables related with these deletions. Finally, the impact of these deletions in RDT results was analysed combining reported information about RDT sensitivity and deletion prevalences.

**Results:**

49 articles were included for the systematic review and 37 for the meta-analysis, 13 of them placed in Africa. Study design differs significantly, especially in terms of population sample and information reported, resulting in high heterogeneity between studies that difficulties comparisons and merged conclusions. Reported prevalences vary widely in all the WHO regions, significantly higher deletion were reported in South-Central America, following by Africa and Asia. *Pfhrp3* deletion is more prevalent (43% in South-Central America; 3% in Africa; and 1% in Asia) than *pfhrp2* deletion (18% in South-Central America; 4% in Africa; and 3% in Asia) worldwide. In Africa, there were not found differences in deletion prevalence by geographical or population origin of samples. The prevalence of deletion among false negatives ranged from 0 to 100% in Africa, but in Asia and South-Central America was only up to 90% and 48%, respectively, showing substantial relation between deletions and false negatives.

**Conclusion:**

The concerning prevalence of *pfhrp2*, *pfhrp3* and *pfhrp2/3* gene deletions, as its possible implications in malaria control, highlights the importance of regular and systematic surveillance of these deletions. This review has also outlined that a standardized methodology could play a key role to ensure comparability between studies to get global conclusions.

**Supplementary Information:**

The online version contains supplementary material available at 10.1186/s12936-021-03812-0.

## Background

Malaria is one of the major challenges for global health. Indeed, during 2019, around 229 million cases were reported worldwide and there were 409,000 deaths. *Plasmodium falciparum* caused the majority of cases and deaths by malaria, followed by *Plasmodium vivax* (2.8% of cases). Africa, where the prevalent species is *P. falciparum*, reported more than 94% of cases and deaths [1].

Prompt and accurate diagnosis is essential for malaria control as it allows effective and timely treatment [2]. Point-of-care testing is a good option in this regard, especially in resource-limited settings, which is typically the case for the majority of endemic malaria regions [3, 4]. The use, variety and quality of malaria rapid diagnostic tests (RDTs) have increased significantly during the last 10 years and they are currently the preferred field diagnostic test for malaria [5]. The majority of RDTs are based on detecting HRP2 (histidine-rich protein 2), a specific protein from *P. falciparum* encoded by the *pfhrp2* gene [6]. However, in addition to detecting HRP2, there are also reports of cross-reactions with HRP3, a structural homologue of HRP2 encoded by the *pfhrp3* gene [7]. As such, RDT may detect both proteins.

Previous studies have demonstrated the efficacy and advantages of RDT compared to other diagnostic techniques, including microscopy and PCR [8–10]. However, its performance has been threatened by the detection of parasites lacking the *pfhrp2* and *pfhrp3* genes since 2010, when these deletions were described for first time [11, 12]. Failure of RDT might also be caused by different factors, such as parasite density, although the deletion of one or both of these genes is considered to be the principal cause of false negatives [13, 14]. Some studies have also suggested that the genetic diversity in *pfhrp2* and *pfhrp3* genes could influence RDT results [15, 16].

It is particularly important to pay special attention to the specificity of diagnostic tests in order to identify people infected. Following a treatment based on diagnostic strategy, a wrong diagnosis means that the patient will not receive the appropriate treatment in time, and could prove fatal [17].

The first evidence of parasites carrying *pfhrp2* and *pfhrp3* gene deletions was reported in South America [18]. Since then, several studies have described these gene deletions in greater proportions across different countries in South and Central America [19, 20], Sub-Saharan Africa [21–23] and Asia [24, 25]. This shows the worldwide spread of parasites with such deletions, with Africa playing a particularly critical role as its high prevalence of malaria caused by *P. falciparum* increases the consequences of these deletions for public health.

A deletion prevalence of 5% in these genes has been defined by the World Health Organization (WHO) as the minimum prevalence for changing the RDT kit, as prevalences higher than 5% could threaten the effectiveness of the test and affect public health guidelines for malaria control [26].

The selection and spread of *pfhrp2* and *pfhrp3* gene deletions may be related to different factors, such as national malaria prevalence or frequencies of people seeking treatment [27], as well as to individual variables, such as age (younger age) [17] or type of symptoms [28].

Deletions are commonly detected in monoclonal infections, which are more common in low prevalence situations [29], during the low malaria transmission season and in countries with a decreasing prevalence of malaria [30, 31]. As such, the spread of these deletions will also partially depend on the epidemiology of the disease in each country. In that context, increasing the understanding of *pfhrp2* and *pfhrp3* gene deletions could play a key role in ensuring the efficacy of strategies for malaria control adapted to each particular region. Moreover, the use of meta-analysis may increase the accuracy of the results found in previous studies.

This paper is an original review and meta-analysis assessing the principal issues of study design and methodological workflow of studies addressing *pfhrp2* deletion and the prevalence status of *pfhrp2* and *pfhrp3* gene deletions worldwide, with special focus on their distribution in Africa. Moreover, this study assesses the possible impact of these deletions in RDT effectiveness.

## Methods

This systematic review and meta-analysis was conducted according to the Preferred Reporting Items for Systematic Reviews and Meta-Analysis (PRISMA) guidance (http://prisma-statement.org/). The PRISMA checklist is provided in Additional file 1.

### Review question

The aim of the study was to review the scientific literature characterizing the deletion of *pfhrp2*, *pfhrp3* and double deletion *pfhrp2* and *3* (*pfhrp2/3*) genes during the last 10 years worldwide. The review sought (1) to assess the main methodological aspects in the study of *pfhrp2*, *pfhrp3* and *pfhrp2/3* gene deletions; (2) to gather the epidemiological information reported for *pfhrp2*, *pfhrp3* and *pfhrp2/3* gene deletions worldwide; (3) to assess the epidemiological information reported for these genes in Africa; and (4) to evaluate the impact of these deletions on the results of PfHRP2-based malaria RDTs.

### Search strategy and data sources

A systematic electronic search was conducted in MEDLINE (PubMed), Scopus and Cochrane, as well as a manual search, in April 2020. The search terms used included “*hrp2*”, “*pfhrp2*”, “*hrp3*”, “*pfhrp3*”, “mRDT” and “*malaria rapid diagnostic test*”. These terms were combined using the Boolean logical operator “OR”. The search was restricted to articles published since January 2010.

### Selection of studies

Two members of the research group (IMF and PB) screened all articles using the eligibility criteria of the review independently and discussed the discrepancies. Firstly, the articles were screened by title and abstract, and finally by full text. All the inclusion and exclusion criteria used in this review are indicated in Table 1. Data analysis differed by specific objective, thus the inclusion criteria applied for each one also varied.

**Table 1:** Inclusion and exclusion criteria by analysis

Analysis where that criteria was applied			Inclusion criteria	Exclusion criteria
Meta-analysis and subgroup analysis	Meta-analysis (quantitative-synthesis)	Qualitative description	Original articles written in English Published and peer-reviewed articles between January 2010 and April 2020 Addressed the status of *pfhrp2*, *pfhrp3* and/or *pfhrp2/3* deletions	It had been written in language different to English Published before 2010 Field isolates were not included Major bias had been detected
			Primary data on *pfhrp2/3* deletion Molecular methodologies to detect deletions Quality of Cross-sectional study score ≥ 5 according to The Joanna Briggs Institute (JBI) (Munn Z, 2017) Sample size of ≥ 30	Year of samples had not been reported Data from more than 3 years are combined
			Placed in Sub-Saharan Africa It included malaria samples from population with different ages, not only children	

### Data management

The following data, summarized in an extraction table, were extracted from each study: author, title, country where samples were obtained, study design, sample characteristics, year of study collection, diagnosis of malaria and confirmation method, information about PfHRP2-RDT, methodological aspects of *pfhrp2/3* gene deletion detection, prevalence of *pfhrp2*, *pfhrp3* and flanking region deletions among all *P. falciparum* cases and among false negative PfHRP-based RDT results, and main conclusions of each study.

### Qualitative synthesis

The descriptive summaries were structured by including all the information in an Excel spreadsheet, sorted by research topic. A qualitative narrative synthesis of findings was carried out in order to assess the main methodological aspects of the study of *pfhrp2* and *pfhrp3* gene deletions (first aim of this study).

### Meta-analysis

The meta-analysis was performed with the studies that met the inclusion criteria and analysed the prevalence of deletion. Prevalence was first calculated for all *P. falciparum* samples included in each study depending on the WHO region, then for studies in Africa (origin of samples) and finally for RDT false-negatives. The prevalence of articles that analysed deletions only among disruptive samples (RDT false-negative and other diagnostic method-positive samples) was included in the general results section using the total population as denominator.

### Statistical analysis

The pooled value of prevalence was established using DerSimonnian-Laird (DL) random effect model applied to proportions [32]. A random model was applied due to the differences between the studies included in terms of study design. In the analysis, the inverse variance method was used to determine the relative weight of each study and logit transformation.

Heterogeneity between studies was calculated using the I^2^ statistic, which describes the percentage variability due to variation between studies. I^2^ represents a measure of inconsistency, and I^2^ > 80% was considered to indicate considerable heterogeneity. To assess publication bias, contour-enhanced funnel plots were generated to study whether small studies with small effects were missing and to visualize the statistical significance of the studies included. These plots included all studies, representing their standard error and their effect. An asymmetry found in the plot could mean publication bias, as studies with more standard error reported greater effects. Additionally, the asymmetry of the funnel plot was tested using the Egger test, and possible p-hacking was tested by performing a P-curve analysis [33].

The meta-analysis was performed using the R 4.0.0 software package to analyse the deletion prevalence data extracted. The meta [34], metafor [35] and dmetar [36] R packages were used.

### Meta-analysis focussed on Africa: subgroup analysis

In order to assess the epidemiological status of *pfhrp2* and *pfhrp3* deletions in Africa by region, only studies carried out in Africa that followed more stringent inclusion criteria were used to perform a subgroup meta-analysis. These studies were analysed independently according to the origin of samples (health facilities or from general population). For the subgroup analysis, a random-effects model was applied within and between subgroups. This model was applied because the data extracted from articles could not be considered to be representative of each African region.

In order to control the between-study heterogeneity in the subgroup analysis, a more in-depth assessment of this heterogeneity was obtained from an influential analysis and a search for outliers. The results of this influential analysis were illustrated with a Baujat Plot calculated using the leave-one-out strategy. Studies whose confidence interval did not overlap with the confidence interval of the pooled prevalence were considered outliers. The results of both analyses were then combined, and studies that overly contributed to heterogeneity and had been identified as outliers were excluded from the subgroup analysis. Once the heterogeneity had been decreased, the analysis was performed independently for each subgroup.

## Results

### Results of systematic review

#### Selection of studies

**Fig. 1:**
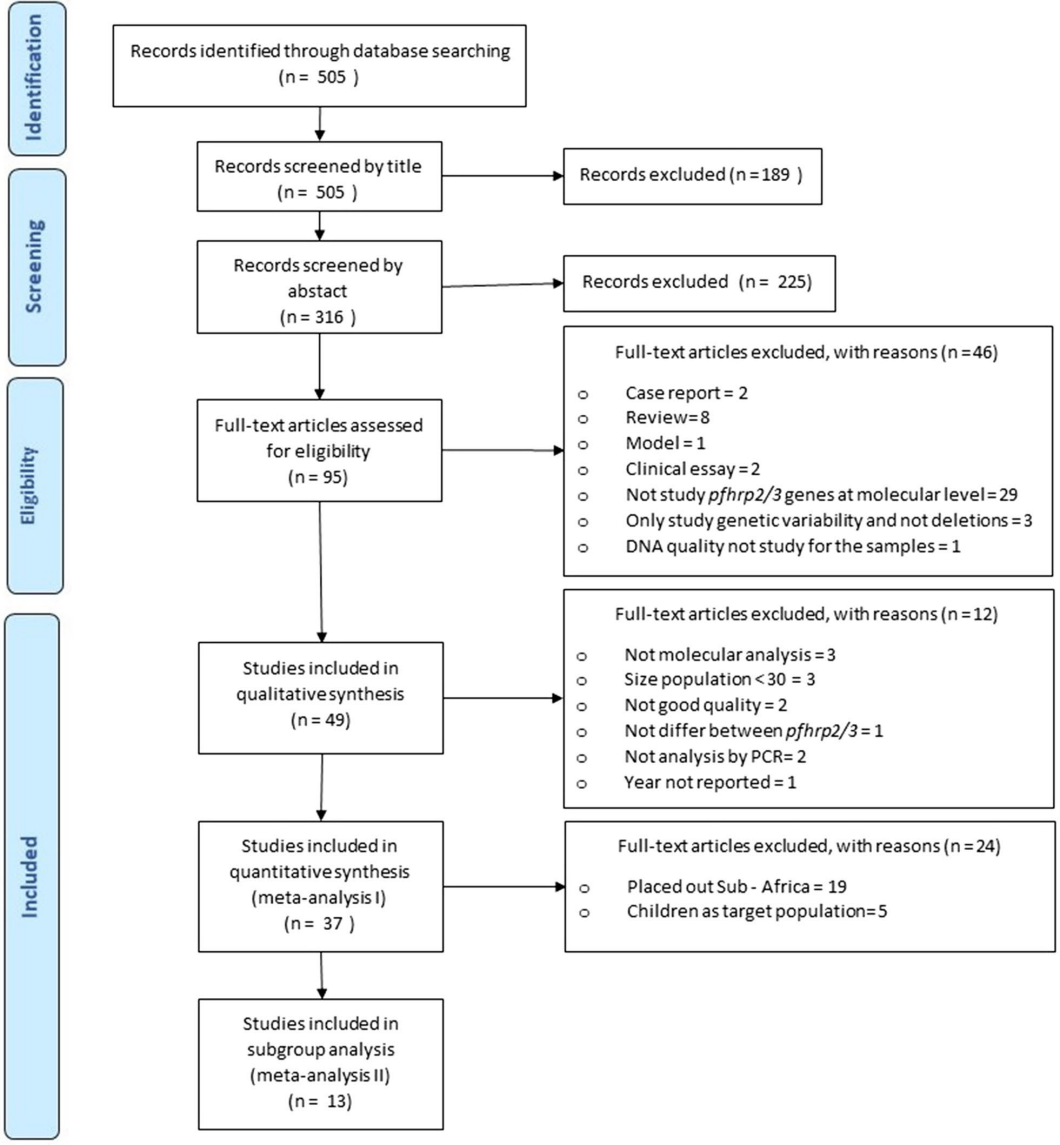
PRISMA flow diagram with the selection and inclusion process

The initial search yielded 505 articles, 95 of which were found to be eligible after title and abstract screening. After full text review, 46 of these were excluded because they did not meet the inclusion criteria. Finally, 49 articles were included in the systematic review for qualitative synthesis. Out of these 49 articles, 37 were included in the quantitative synthesis and 13 in the in-depth meta-analysis with subgroup analysis (Fig. 1).

#### Characteristics of studies included: population and setting

All the studies included were cross-sectional, with 22 being conducted in Africa, 12 in South America, seven in Asia, four in Central America and one in Oceania. A further study combined data from different WHO regions [15]. A total of 24 studies reported data from several regions within a country and eight studies combined and compared data from different countries [12, 15, 37–41]. The remaining studies did not specify the reporting of data from different regions.

Twenty five of the 49 studies included used only malaria-confirmed field samples. Of the studies conducted with population sampling (samples with and without malaria infection), 18 targeted the general population and 10 included only the symptomatic population suspected of malaria. The other 24 studies included samples from the general population, combining infected and non-infected samples. A total of six studies focused on children, with two of these involving symptomatic children (Table 2).

**Table 2:** Characterization of articles included which study molecular deletions on *pfhrp2/3*

First author	Country	Samples’ year	Collection season	Study population	Methodologies
Abdallah [19]	Honduras	2008–09	Both or annual	Malaria confirmed field samples	PCR, SSR/STR
Okoth [39]	Suriname and Guyana	2010–11	High transmission	Malaria confirmed field samples	PCR, SSR/STR
Akinyi [57]	Peru	1998–05	High transmission	Malaria confirmed field samples	PCR, SSR/STR
Amoah [74]	Ghana	2014–15	Low transmission	Population sampling among children, asymptomatic	PCR
Atroosh [48]	Yemen	2012	High transmission	Population sampling among all ages, symptomatic	PCR, Seq
Baker [15]	18 countries^a^	2010	Not reported	Malaria confirmed field samples	PCR, Seq
Baldeviano [59]	Peru	2010–12	Malaria outbreak	Malaria confirmed samples among symptomatic patients at health centre	PCR, SSR/STR
Berhane [14]	Eritrea	2016	High transmission	Population sampling among all ages, from health centre	PCR, SSR/STR
Berzosa [21]	Equatorial Guinea	2013	Both or annual	Population sampling among all ages	PCR, Seq., SSR/STR
Beshir [49]	Kenya	2007–08 and 2014	Not reported	Population sampling among symptomatic at health centre and among children Asymptomatic	PCR, Seq
Bharti [24]	India	2014	High transmission	Population sampling among all ages, symptomatic	PCR, Seq
Deme [38]	Senegal, Mali and Uganda	2001–10	High transmission	Malaria confirmed field samples, from health centre	Seq., SSR/STR
Dong [58]	China	2013–18	Both or annual	Population sampling among all ages, symptomatic	PCR, Seq., SSR/STR
Dorado [20]	Colombia	2003–12	Not reported	Malaria confirmed field samples	PCR, Seq., SSR/STR
Fontecha [41]	Honduras Guatemala and Nicaragua	2011 and 2015	Not reported	Malaria confirmed field samples	PCR
Funwei [69]	Nigeria	2013–14	Both or annual	Population sampling among children, Symptomatic	PCR, Seq
Gamboa [18]	Peru	2003–07 and 2007	Not reported	Malaria confirmed field samples from health facility and population sampling among symptomatic (active case detection)	PCR, Seq., ELISA
Gupta [22]	Mozambique	2010–16	Both or annual	Population sampling among all ages	PCR
Herman [43]	Haiti	2012–14	Both or annual	Population sampling among all ages, symptomatic	PCR
Kobayashi [52]	Zambia	2009–11 and 2015–17	High transmission	Population sampling among all ages	PCR
Koita [28]	Mali	1996	High transmission	Population sampling among all ages and children asymptomatic	PCR
Kozycki [11]	Ruanda	2014–15	Both or annual	Population sampling among all ages, symptomatic at health centre	PCR
Kreidenweiss [55]	Gabon	2017–18	Both or annual	Lab strains and clinical sample	PCR, Seq
Kumar Bharti [71]	India	2014	Both or annual	Malaria confirmed field samples	PCR, Seq
Kumar [42]	India	2010	Both or annual	Malaria confirmed field samples	PCR
Kumar [50]	India	2009–11	Both or annual	Malaria confirmed field samples	PCR, Seq
Laban [54]	Zambia	2008–12	Both or annual	Population sampling among all ages	PCR
Li [45]	China	2011–12	Both or annual	Malaria confirmed field samples	PCR, Seq
Maltha [13]	Peru	2010–11	Both or annual	Malaria confirmed field samples, from health centre	PCR
Menegon [23]	Eritrea	2013–14	Not reported	Population sampling among all ages	PCR
Murillo-Solano [63]	Colombia	1999–09	Both or annual	Malaria confirmed field samples	PCR, SSR/STR
Mussa [61]	Sudan	Not reported	Not reported	Population sampling among all ages, symptomatic	PCR, Seq
Nderu [9]	Kenya	2007–16	Not reported	Malaria confirmed field samples, from symptomatic patients	PCR, Seq
Nderu [60]	Kenya	2016	Both or annual	Malaria confirmed field samples from symptomatic patients	PCR, Seq., SSR/STR
Okoth [44]	Peru	2013	Malaria outbreak	Malaria confirmed field samples from symptomatic patients	PCR, SSR/STR
Owusu [47]	Ghana	2015	Both or annual	Population sampling among children, HIV positives and healthy	PCR
Parr [46]	D.R. of Congo	2013–2014	Both or annual	Population sampling among children, majority asymptomatic	PCR, SSR/STR
Pati [25]	India	2013–16	Both or annual	Population sampling among all ages, symptomatic	PCR, Seq
Plucinski [51]	Angola	2016	High transmission	Population sampling among all ages, at health centre	PCR
Rachid Viana [40]	Brazil and Bolivia	2010–12	Not reported	Malaria confirmed field samples, at health centre	PCR
Ramutton [37]	7 countries^b^	2005–10	Not reported	Population sampling among children with severe malaria	PCR, Seq
Ranadive [53]	Swaziland	2012–15	Both or annual	Population sampling among all ages, symptomatic	PCR
Sáenz [56]	Ecuador	2012–13	Both or annual	Malaria confirmed field samples	
Thomson [12]	Ghana Tanzania and Uganda	2009–10 and 2014–15	High transmission	Malaria confirmed field samples from symptomatic population with all ages, at health facilities and national survey	PCR
Trouvay [73]	French Guiana	2009 and 2011	Both or annual	Malaria confirmed field samples	PCR, Seq., SSR/STR
Willie [30]	Papua New Guinea	2001–03	Both or annual	Population sampling among all ages	PCR, Seq
Willie [62]	Madagascar	2014–15	Both or annual	Population sampling among all ages, mostly asymptomatic	PCR, Seq
Wurtz [16]	Senegal	2009–11	High transmission	Malaria confirmed field samples, at health centre	PCR, Seq

*Seq.* sequencing, *SSR/STR* techniques based on microsatellite analysis

^a^Benin, Burkina Faso, Cameroon, Central African Republic, Gambia, Ghana, Guinea, Kenya, Liberia, Madagascar, Malawi, Nigeria, Niger, Sierra Leone, Sudan, Tanzania, Uganda, Zambia, Papua New Guinea, Solomon Is., East Timor, Vanatu, Brazil, Colombia, Ecuador, Haiti, Honduras, Peru, Santa Lucia, SUriname, Cambodia, China, Indonesia, Malaysia, Myanmar, Philippines, Thailand and Vietnam

^b^Democratic Republic of Congo, Gambia, Kenya, Mozambique, Rwanda, Tanzania and Uganda

#### Methodological aspects of the study of pfhrp2, pfhrp3 and pfhrp2/3 deletions

All studies used molecular techniques to detect *pfhrp2*, *pfhrp3* and *pfhrp2/3* deletions (Table 3). The total sample size included in the studies ranged from 48 [42] to 9317 [43], with a median sample size of 911 [12]. The number of confirmed *P. falciparum* cases included ranged from 4 [44] to 3291 [11], with a median confirmed cases sample size of 169 [30].

**Table 3:** Key points of the methodology to detect *pfhrp2/3* deletions by molecular analysis

First author	Method of malaria diagnosis/confirmation	N total	N cases	N pfhrp	Genes study by molecular analysis (Pfhrp2/Pfhrp3/pfhrp2/3/Flanking genes)	Method to test DNA quality	Elimination for parasitaemia ≤ 5 p/µL
Abdallah [19]	PCR	68	68	68	*pfhrp2*, *pfhrp3*, *pfhrp2/3*, *flanking genes*	PCR	NA
Okoth [39]	Microscopy/PCR	203	175	175	*pfhrp2*, *pfhrp3*, *pfhrp2/3*, *flanking genes*	PCR, qPCR	No
Akinyi [57]	PCR	188	188	188	*pfhrp2*, *flanking genes*	PCR	NA
Amoah [74]	RDT, microscopy/PCR	558	288	288	*pfhrp2*, *pfhrp*, *pfhrp2/3*	PCR	NA
Atroosh [48]	RDT, microscopy	622	189	189	*pfhrp2*	PCR	NA
Baker [15]	RDT, microscopy	458	458	458	*pfhrp2*, *pfhrp3*	NA	NA
Baldeviano [59]	Microscopy/PCR	210	54	54	*pfhrp2*	PCR	NA
Berhane [14]	RDT, microscopy/PCR	51	50	50	*pfhrp2*, *pfhrp3*, *pfhrp2/3*, *flanking genes*	PCR	NA
Berzosa [21]	RDT/PCR	1724	1724	122	*pfhrp2*, *pfhrp3*, *pfhrp2/3*	PCR	NA
Beshir [49]	RDT, microscopy/PCR	274	131	131	*pfhrp2*, *pfhrp3*, *pfhrp2/3*, *flanking genes*	qPCR	Yes
Bharti [24]	RDT, microscopy/PCR	1521	1521	50	*pfhrp2*, *pfhrp3*, *pfhrp2/3*, *flanking genes*	qPCR	No
Deme [38]	PCR	74	74	NA	NA	PCR	NA
Dong [58]	Microscopy/PCR	306	306	306	*pfhrp2*	PCR	NA
Dorado [20]	RDT, microscopy/PCR	374	365	365	*pfhrp2*, *pfhrp3*, *pfhrp2/3*, *flanking genes*	PCR	NA
Fontecha [41]	Microscopy/PCR	128	128	128	*pfhrp2*, *pfhrp3*, *pfhrp2/3*, *flanking genes*	PCR	NA
Funwei [69]	RDT, microscopy/PCR	511	340	66	*pfhrp2*, *pfhrp3*, *pfhrp2/3*	PCR	NA
Gupta [22]	RDT, microscopy/PCR	9124	1162	69	*pfhrp2*, *pfhrp3*	PCR, qPCR	Yes
Gamboa [18]	RDT, microscopy/PCR	157	157	157	*pfhrp2*, *pfhrp3*, *pfhrp2/3*, *flanking genes*	PCR	NA
Herman [43]	RDT/PCR	9317	2695	7	*pfhrp2*	PCR	NA
Kobayashi [52]	RDT, microscopy/PCR	5167	1189	36	*pfhrp2*	qPCR	Yes
Koita [28]	RDT, microscopy	723	480	37	*pfhrp2*	PCR	NA
Kozycki [11]	RDT, microscopy	8757	3291	370	*pfhrp2*	PCR	Yes
Kreidenweiss [55]	RDT/PCR	200	200	95	*pfhrp2*	PCR, qPCR	Yes
Kumar Bharti [71]	RDT/PCR	1392	1392	1392	*pfhrp2*, *pfhrp3*, *pfhrp2/3*	PCR	NA
Kumar [42]	RDT, microscopy/PCR	48	48	48	*pfhrp2*, *pfhrp3*, *pfhrp2/3*, *flanking genes*	PCR	NA
Kumar [50]	RDT/PCR	140	48	48	*pfhrp2*, *pfhrp3*	qPCR	No
Laban [54]	RDT, microscopy/PCR	3292	61	61	*pfhrp2*	PCR, qPCR	Yes
Li [45]	RDT/PCR	97	97	97	*pfhrp2*, *pfhrp2/3*	qPCR	No
Maltha [13]	RDT, Microscopy/PCR	182	74	74	*pfhrp2*, *pfhrp3*, *pfhrp2/3*	qPCR	No
Menegon [23]	Microscopy/PCR	144	144	144	*pfhrp2*, *pfhrp3*, *pfhrp2/3*, *flanking genes*	PCR	NA
Murillo Solano [63]	RDT, microscopy/PCR	115	100	100	*pfhrp2*, *pfhrp3*, *pfhrp2/3*, *flanking genes*	NA	NA
Mussa [61]	RDT/PCR	59	26	26	*pfhrp2*	PCR	NA
Nderu [9]	PCR	400	400	400	*pfhrp2*, *pfhrp3*	PCR	NA
Nderu [60]	RDT, microscopy/PCR	80	80	80	*pfhrp2*	PCR	NA
Owusu [47]	RDT, microscopy/PCR	401	62	8	*pfhrp2*, *pfhrp3*, *pfhrp2/3*, *flanking genes*	PCR	NA
Okoth [44]	Microscopy/PCR	4	4	4	*pfhrp2*, *pfhrp3*, *pfhrp2/3*	PCR	NA
Parr [46]	RDT, microscopy/PCR	7137	2752	2752	*pfhrp2*, *pfhrp3*, *pfhrp2/3*	qPCR	No
Pati [25]	RDT, Microscopy/PCR	1058	384	384	*pfhrp2*, *pfhrp3*, *pfhrp2/3*, *flanking genes*	PCR	NA
Plucinski [51]	RDT/PCR	1267	458	5	*pfhrp2*, *pfhrp3*, *pfhrp2/3*	qPCR	No
Rachid Viana [40]	Two RDT, Microscopy	223	223	223	*pfhrp2*, *pfhrp3*, *pfhrp2/3*, *flanking genes*	PCR	NA
Ramutton [37]	RDT/PCR	3826	77	77	*pfhrp2*, *pfhrp3*	PCR, qPCR	Yes
Ranadive [53]	RDT/PCR	1353	162	9	*pfhrp2*	PCR, qPCR	Yes
Sáenz [56]	Microscopy/PCR	32	32	32	*pfhrp2*, *pfhrp3*, *pfhrp2/3*	PCR	NA
Thomson [12]	RDT, microscopy/PCR	911	718	411	*pfhrp2*, *pfhrp3*, *pfhrp2/3*	PCR, qPCR	Yes
Trouvay [73]	RDT, microscopy/PCR	359	221	221	*pfhrp2*, *pfhrp3*	PCR	NA
Willie [30]	RDT/PCR	169	169	137	*pfhrp2*	PCR	NA
Willie [62]	RDT, microscopy/PCR	260	73	73	*pfhrp2*	PCR	NA
Wurtz [16]	RDT, microscopy/PCR	136	125	125	*pfhrp2*, *pfhrp3*	qPCR	No

*N total* total of samples included in the study, *N cases* total of *P. falciparum* confirmed cases included in the study, *N pfhrp* no. of samples included for molecular analysis (PCR), *pfhrp2/3* double deletion *pfhrp2* + *pfhrp*, *NA* not applicable

Some studies performed the molecular analysis of *pfhrp2/3* deletions only on a subset of the total sample size. Ten studies only performed a molecular analysis for discordant samples with an HRP2-based RDT negative result and malaria PCR positive result [16, 21, 22, 24, 25, 42, 45–47].

Regardless of the origins of the sample size, a diagnosis of malaria was performed in all studies, and in the majority (n = 45) this diagnosis was confirmed using another diagnostic method: 27 studies used PCR and microscopy for the diagnosis, 14 PCR only, three microscopy only [11, 28, 48] and one microscopy and another malaria RDT [40]. In addition, 16 studies confirmed the density of parasite DNA by quantitative PCR to confirm that a low density was not the cause of the false negative [12, 13, 16, 22, 24, 37, 39, 45, 46, 49–55]. Moreover, samples included in 38 studies were tested with HRP2-based RDT, whereas 11 studies did not include a diagnostic by RDT [9, 19, 23, 38–41, 44, 56–59].

Deletion of the *pfhrp2* gene was analysed in all studies included in the review, 14 studies only included the analysis of this gene [11, 28, 30, 31, 43, 48, 52–55, 58, 60–62]. Deletion of the *pfhrp3* gene was studied in 29 articles and double deletion of the *pfhrp2* and *pfhrp3* genes was assessed in 26 articles. The deletion was identified by PCR in all studies except one (n = 48). Additionally, 15 articles tested the presence of deletions on *pfhrp2* and *pfhrp3* flanking genes [14, 18–20, 23–25, 39–42, 47, 49, 57, 63].

### Results of quantitative analysis of the prevalence of deletions

The meta-analysis performed for *pfhrp2*, *pfhrp3* and *pfhrp2/3* deletions reported a high between-study heterogeneity (I^2^ ≥ 90%), which meant that it was difficult to obtain statistically significant results. As such, a meta-analysis was carried out as a quantitative summary of the published studies in the WHO region. To explore the potential sources of heterogeneity, a subgroup analysis was also carried out, although no significant results were obtained (Additional file 2).

### Prevalence of *pfhrp2*, *pfhrp3* and *pfhrp2/3* deletions

#### Prevalence of *pfhrp2* deletion

A total of 37 studies were included in the meta-analysis based on the eligibility criteria.

**Fig. 2:**
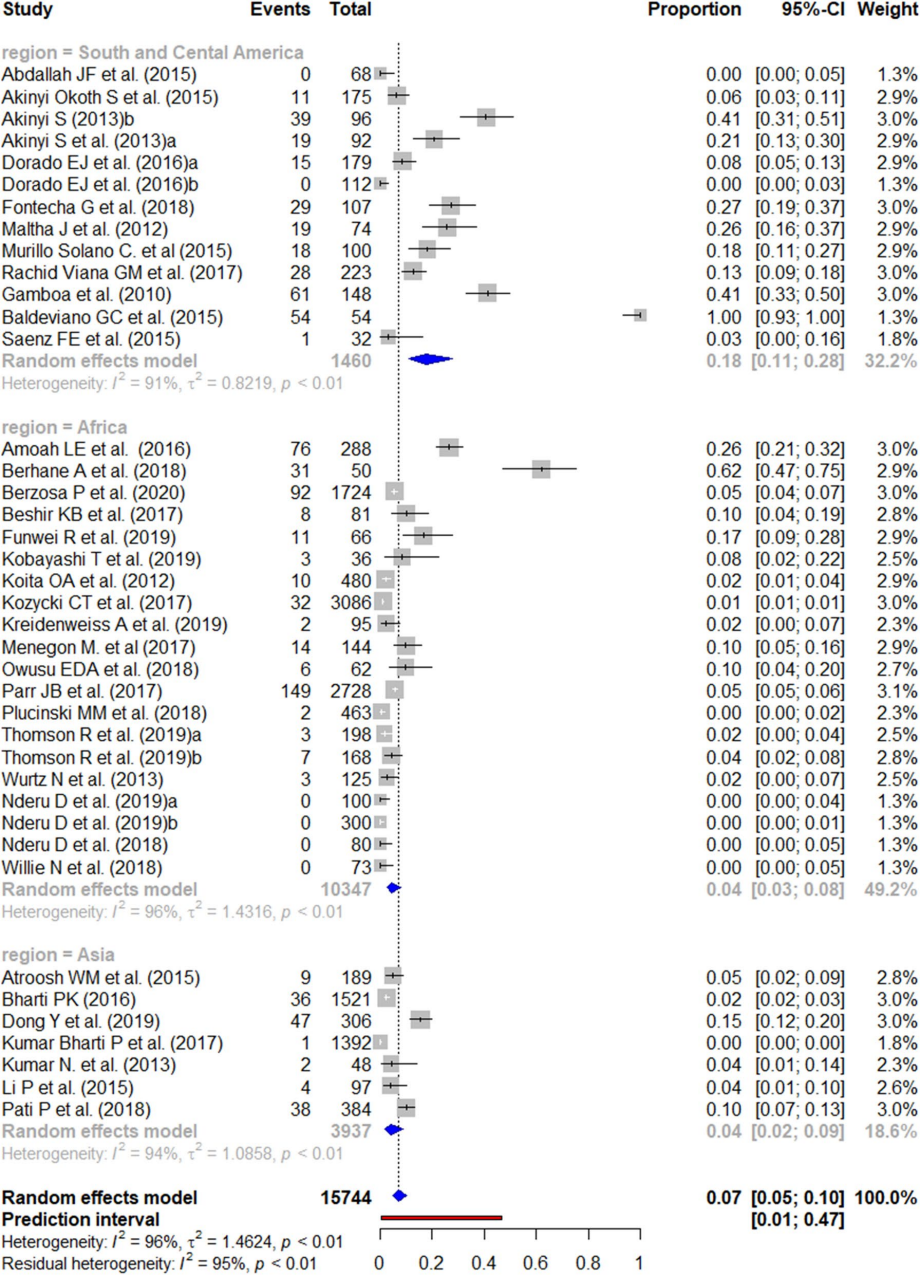
Forest plot showing the prevalence of *pfhrp2* deletions worldwide and by WHO regions. Each grey square represents one study (size proportional to relative weight) and black lines represent the effect and its confidence interval. Pooled prevalences are represented as blue diamonds for each WHO region and worldwide and the prediction interval is represented in red

**Fig. 3:**
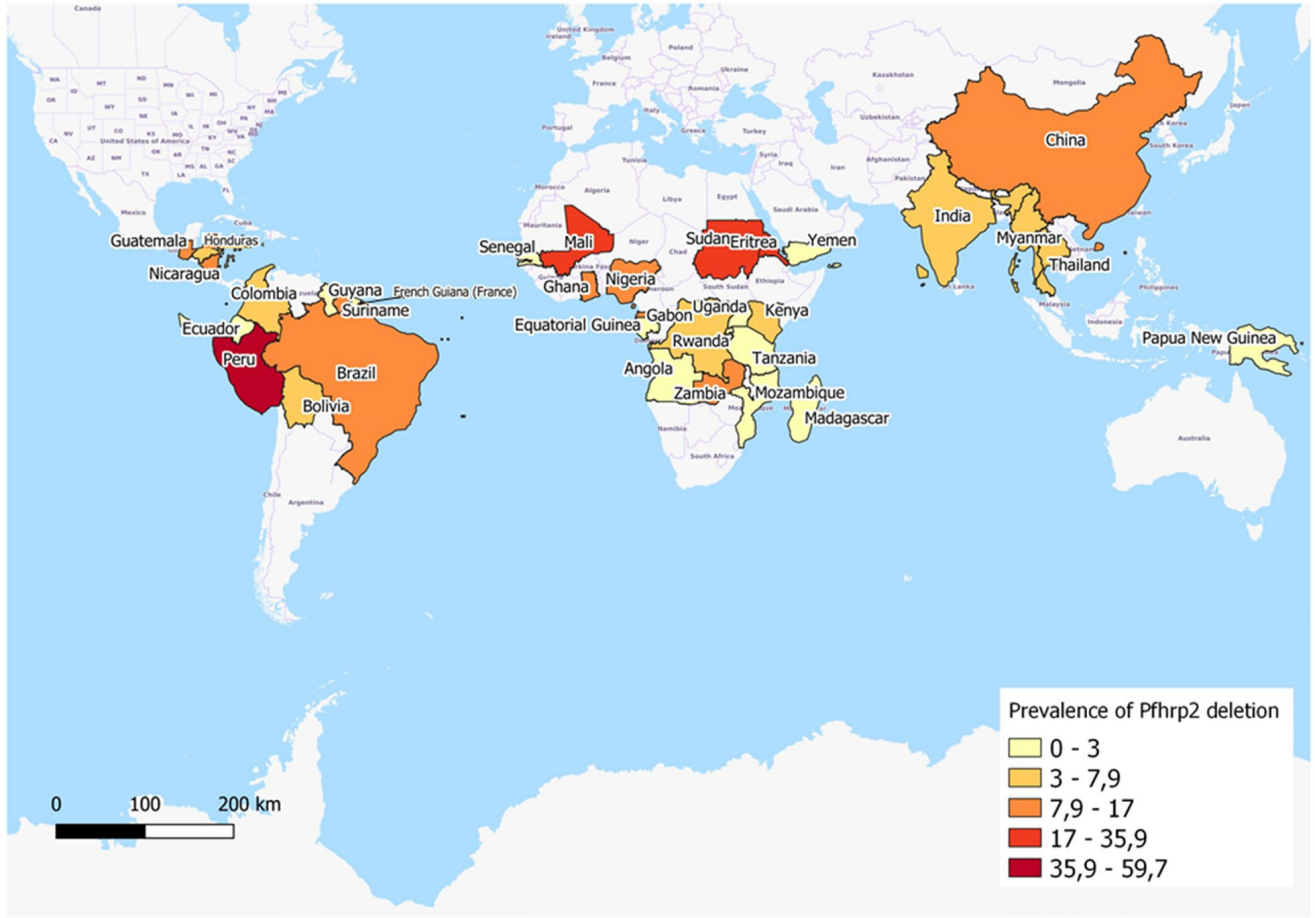
Geographical distribution for the reported prevalence of *pfhrp2* deletions by country. Prevalence by country has been calculated using the mean prevalence of studies undertaken in each country. The representation was produced using jenks (natural intervals)

The reported prevalence of the *pfhrp2* deletion varied from 0 to 100% (Figs. 2 and 3). Those studies reporting the highest prevalence (100%) were carried out in South and Central America [59], followed by Africa (62.0% in Eritrea) [14]. There were seven studies from the three WHO regions reporting an absence of deletions. The mean pooled prevalence for each WHO region was 18% in South and Central America, 4% in Africa, and 3% in Asia. No significant publication bias was found amongst the studies included (Additional file 3).

#### Prevalence of *pfhrp3* deletion

**Fig. 4:**
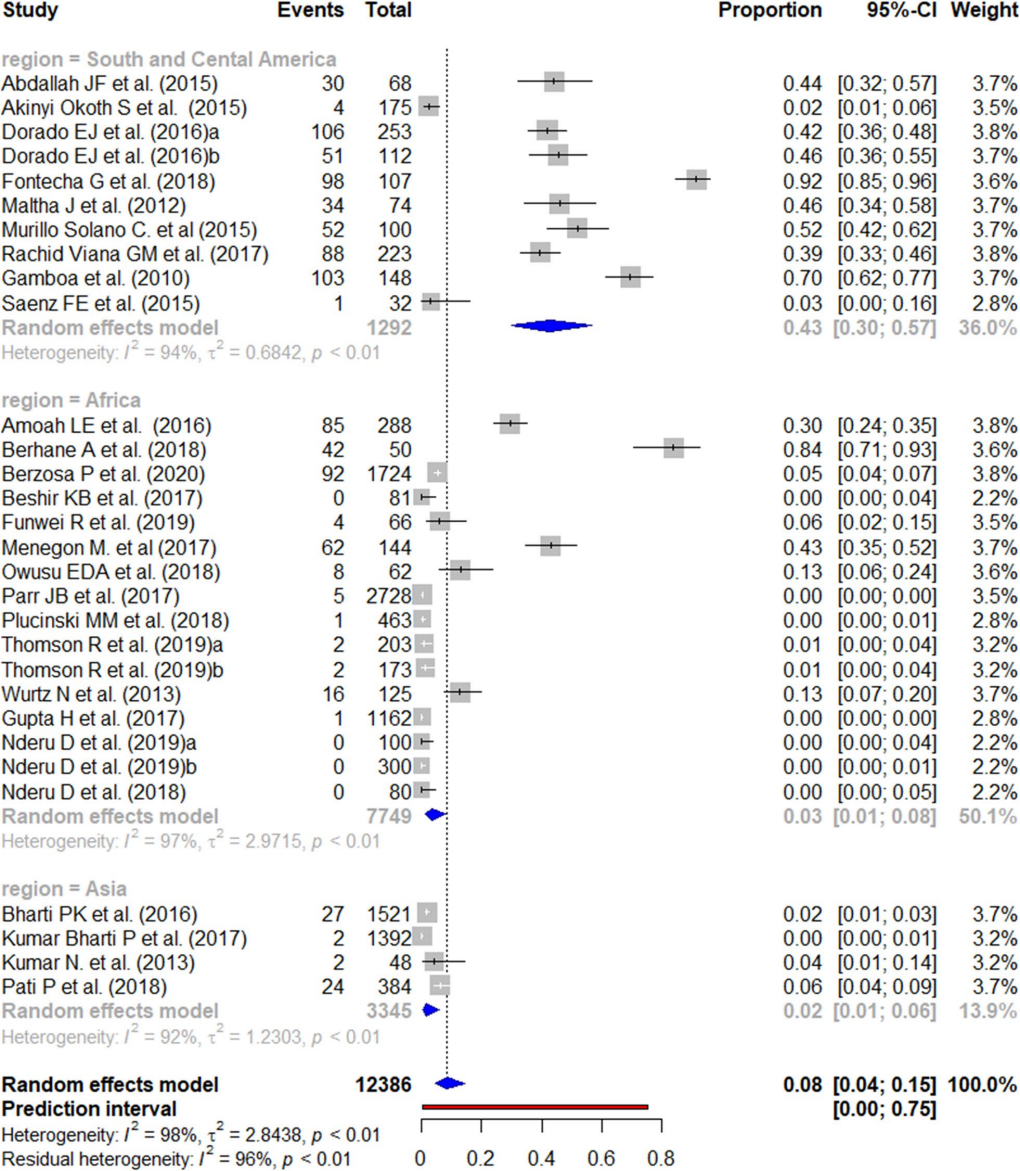
Forest plot showing the prevalence of *pfhrp3* deletions worldwide and by WHO regions. Each grey square represents one study (size proportional to relative weight) and black lines represent the effect and its confidence interval. Pooled prevalences are represented as blue diamonds for each WHO region and worldwide and the prediction interval is represented in red

**Fig. 5:**
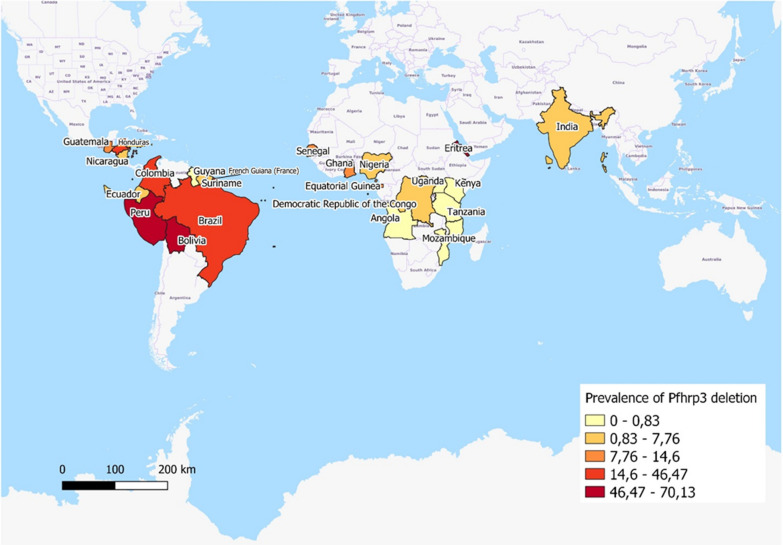
Geographical distribution for the reported prevalence of *pfhrp3* deletions by country. Prevalence by country has been calculated using the mean prevalence of studies undertaken in each country. The representation was produced using jenks (natural intervals)

A total of 30 studies were included in the meta-analysis according to the eligibility criteria. The worldwide prevalence of *pfhrp3* deletion ranged from 0 to 92%. Four studies, all from Africa, reported the absence of this deletion. In contrast, the highest prevalence of mutation was reported in South and Central America, where the prevalence ranged from 2 to 91%. The pooled prevalence of *pfhrp3* deletions by WHO regions was 43% in South and Central America; 3% in Africa; and 1% in Asia (Figs. 4 and 5). The risk of publication bias was not significant (Additional file 4).

#### Prevalence of *pfhrp2* and *pfhrp3* double deletion

**Fig. 6:**
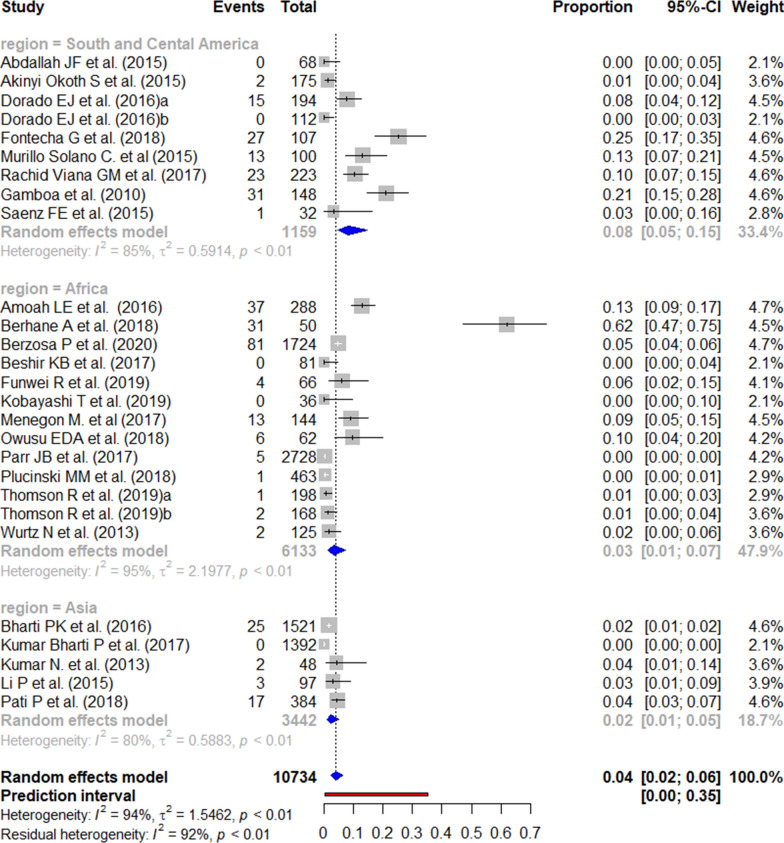
Forest plot showing the prevalence of *pfhrp2/pfhrp3* double deletion worldwide and by WHO regions. Each grey square represents one study (size proportional to relative weight) and black lines represent the effect and its confidence interval. Pooled prevalences are represented as blue diamonds for each WHO region and worldwide and the prediction interval is represented in red

A total of 23 studies were included in the meta-analysis for the *pfhrp2/3* double deletion. The global pooled prevalence of reported *pfhrp2/pfhrp3* double deletions was 4%, with the prevalence reported by WHO region ranging from 0 to 25% in South and Central America, from 0 to 62% in Africa, and 0% to 4% in Asia (Fig. 6).

The risk of publication bias was not significant (Additional file 5).

### Meta-analysis of *pfhrp2*, *pfhrp3* and *pfhrp2/3* deletions in Africa

#### Pooled prevalence and subgroup analysis of *pfhrp2*, *pfhrp3* and *pfhrp2/3* deletions among samples from health facilities and the general population in Africa

**Fig. 7:**
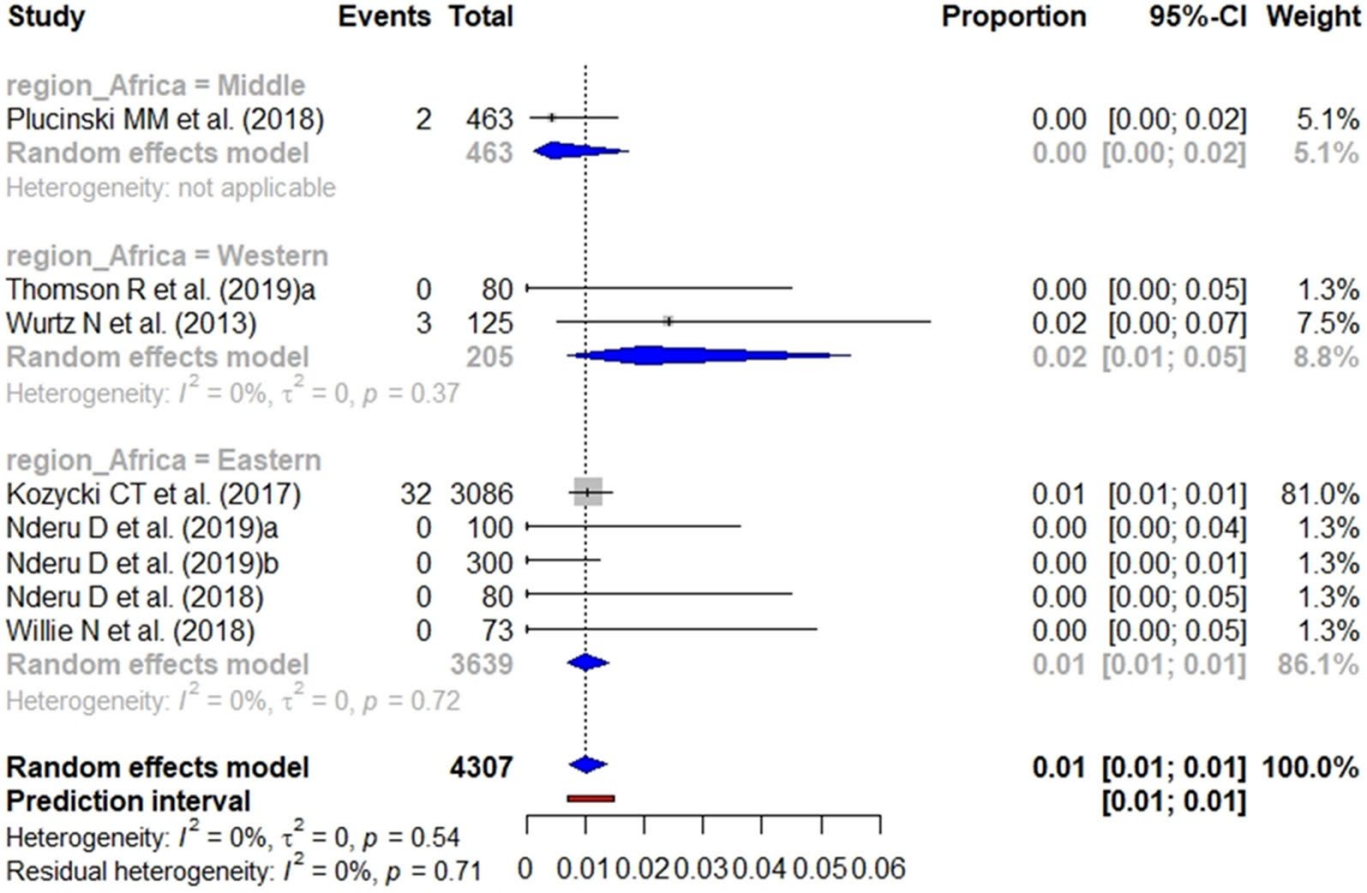
Forest plot showing the prevalence of *pfhrp2* deletion among studies carried out in health facilities. Each grey square represents one study (size proportional to relative weight) and black lines represent the effect and its confidence interval. Pooled prevalences are represented as blue diamonds for each Africa geographical region

**Fig. 8:**
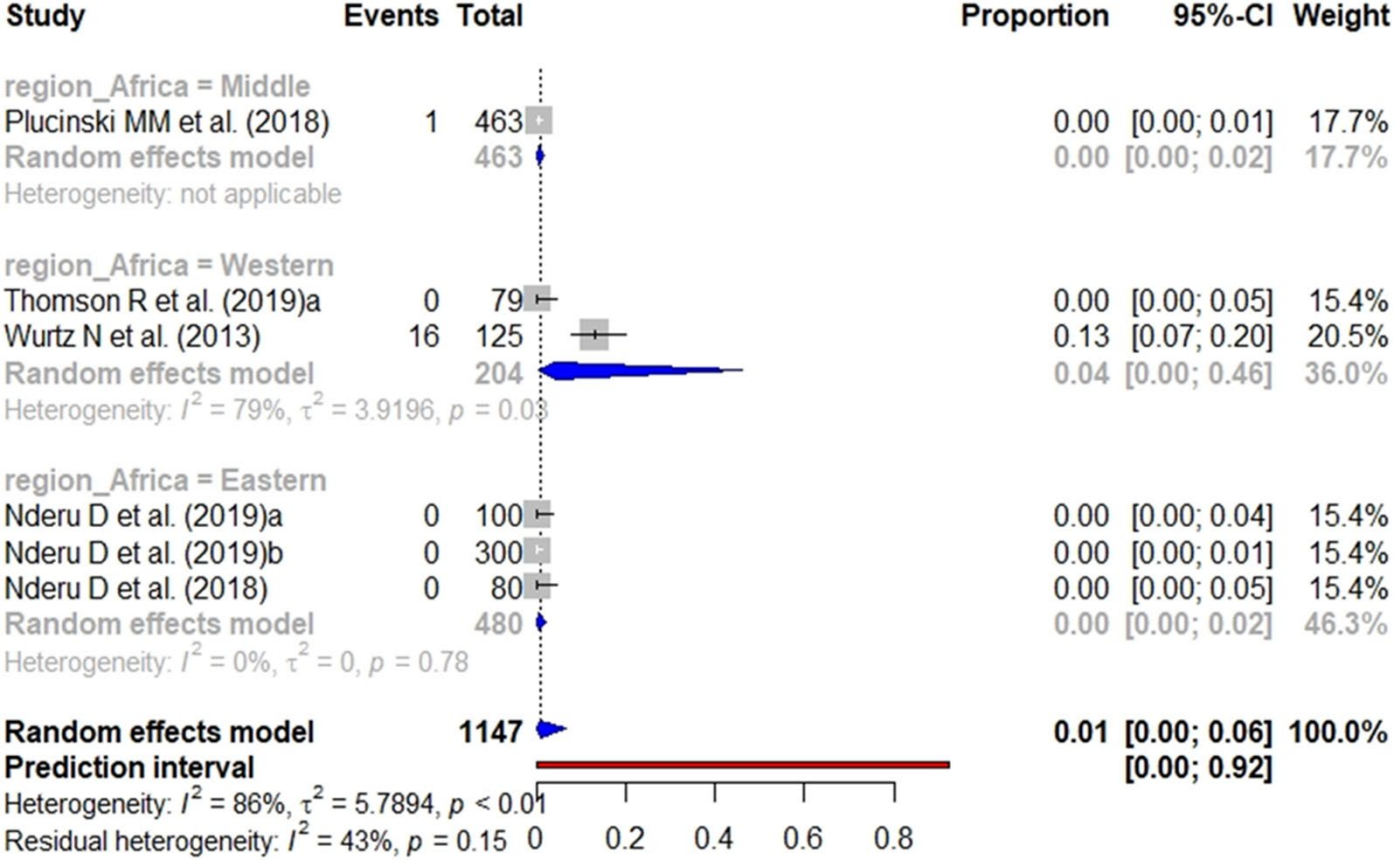
Forest plot showing the prevalence of *pfhrp3* deletion among studies carried out in health facilities. Each grey square represents one study (size proportional to relative weight) and black lines represent the effect and its confidence interval. Pooled prevalences are represented as blue diamonds for each Africa geographical region

**Fig. 9:**
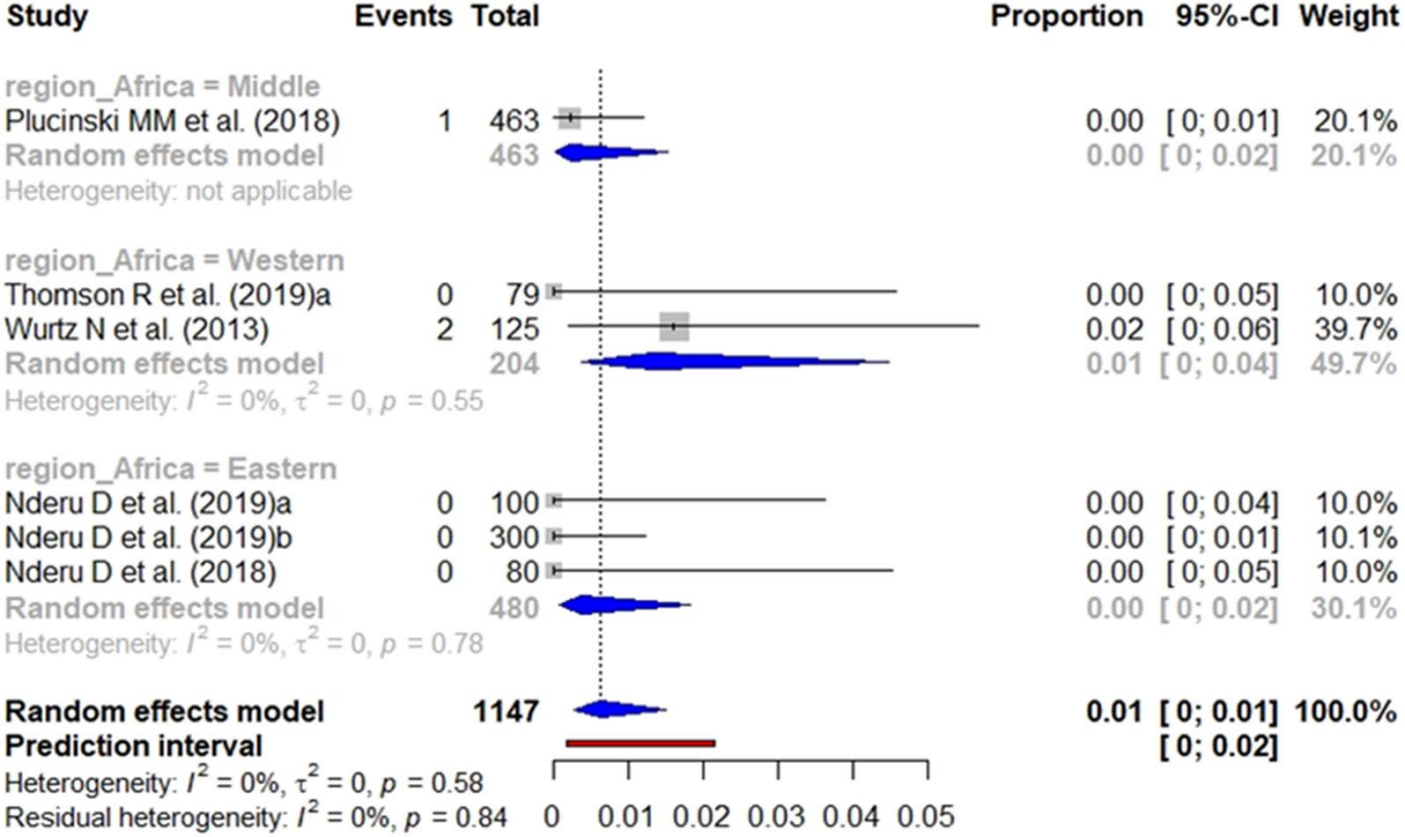
Forest plot showing the prevalence of *pfhrp2/pfhrp3* deletion among studies carried out in health facilities. Each grey square represents one study (size proportional to relative weight) and black lines represent the effect and its confidence interval. Pooled prevalences are represented as blue diamonds for each Africa geographical region

According to the heterogeneity statement for each analysis (Additional files 6, 7, 8), the subgroup analysis for *pfhrp2*, *pfhrp3* and *pfhrp2/3* deletions included 12 (8 from health facilities and 4 from general population), 10 (6 from health facilities and 4 from general population) and 12 studies (6 from health facilities and 6 from general population), respectively. The pooled prevalence of *pfhrp2* (Fig. 7), *pfhrp3* (Fig. 8), and *pfhrp2/3* deletions (Fig. 9) from studies carried out at health facilities was 1%.

The subgroup analysis performed by geographical region in Africa showed a significant difference (p < 0.001) between the aggregate prevalence for the *pfhrp3* single deletion by region, with the highest *pfhrp3* deletion prevalence being found in Western Africa (13%). The difference for the *pfhrp2* single deletion and *pfhrp2/3* double deletion was not significant.

**Fig. 10:**
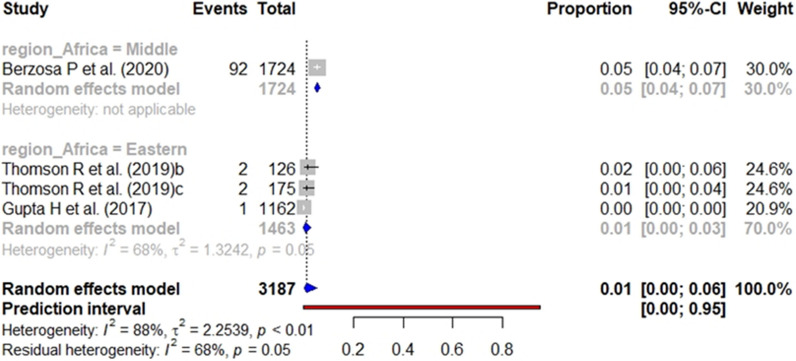
Forest plot showing the prevalence of *pfhrp2* deletion among studies targeting general population. Each grey square represents one study (size proportional to relative weight) and black lines represent the effect and its confidence interval. Pooled prevalences are represented as blue diamonds for each Africa geographical region

**Fig. 11:**
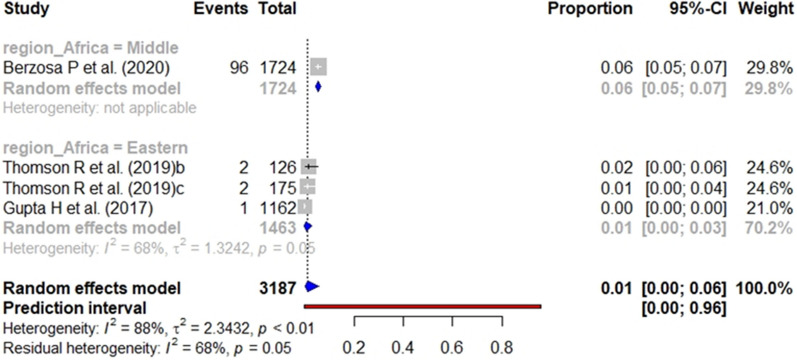
Forest plot showing the prevalence of *pfhrp3* deletion among studies targeting general population. Each grey square represents one study (size proportional to relative weight) and black lines represent the effect and its confidence interval. Pooled prevalences are represented as blue diamonds for each Africa geographical region

**Fig. 12:**
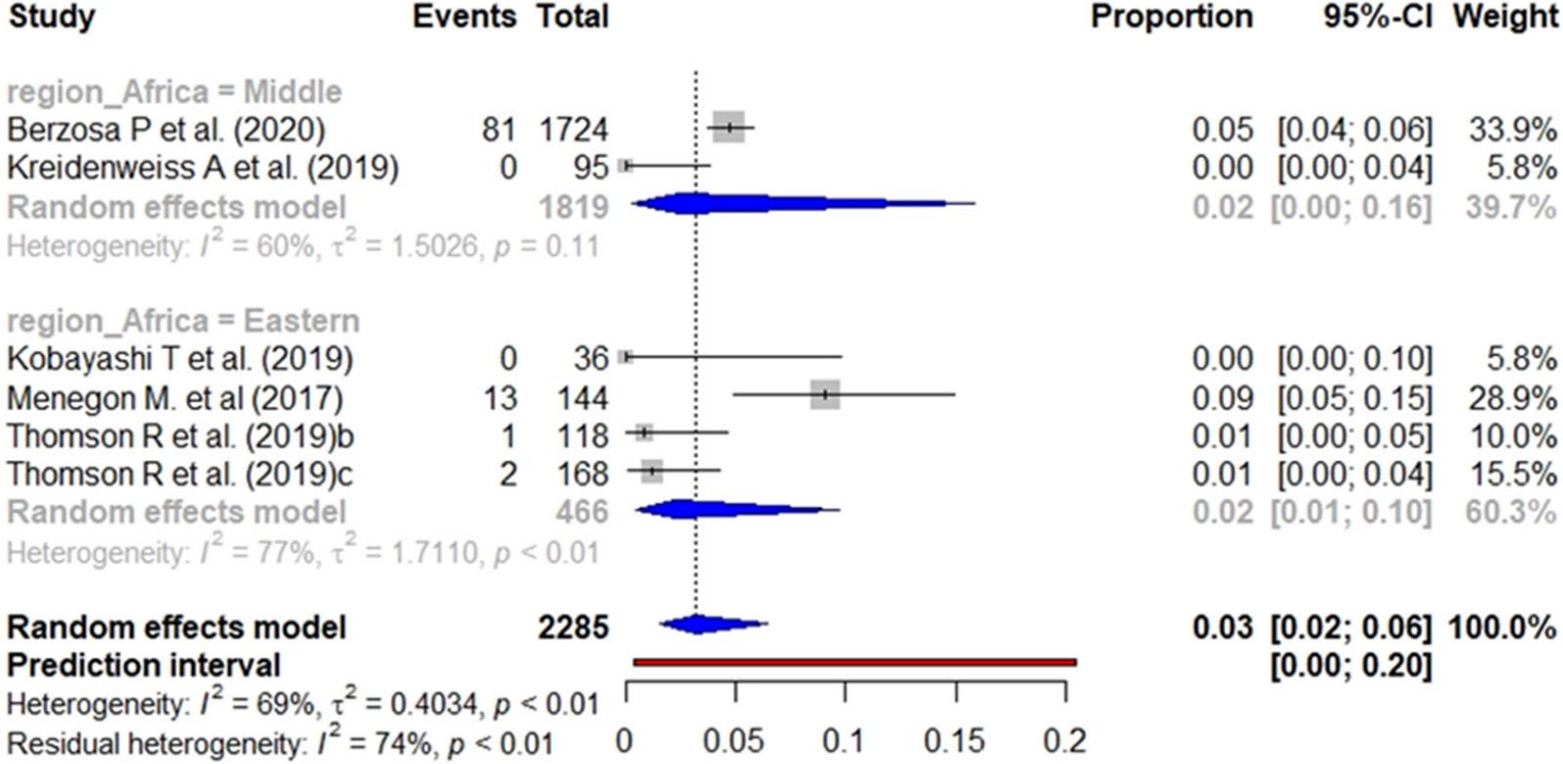
Forest plot showing the prevalence of *pfhrp2* &* pfhrp3* double deletion among studies targeting general population. Each grey square represents one study (size proportional to relative weight) and black lines represent the effect and its confidence interval. Pooled prevalences are represented as blue diamonds for each Africa geographical region

In the case of studies targeting the general population, the pooled prevalence of *pfhrp2* (Fig. 10), *pfhrp3* (Fig. 11), and *pfhrp2/3* deletions (Fig. 12) was 4%, 1% and 3%, respectively. The subgroup analysis by geographical region found no significant differences (p value < 0.001) between the aggregate prevalence for any deletion.

### Impact of *pfhrp2*, *pfhrp3* and *pfhrp2/3* deletions on false-negative RDT results

#### Review of RDT efficacy and *pfhrp2*, *pfhrp3* and *pfhrp2/3* deletions

Published data regarding the efficacy of RDT by study is reported in Table 4. The HRP2-based RDT sensitivity was over 70% for all studies, except for one [14]. As regards the false-negative rate, the highest was 62% and the lowest 2%.

**Table 4:** Reported data about *pfhrp2*, *pfhrp3* and *pfhrp2/3* deletions among RDT false

Study	WHO region	N	HRP2-RDT sensitivity (%)	False negative rate (%)	Negative like-hood ratio	P (%) of *pfhrp2* deletion (%)	P (%) of *pfhrp3* deletion (%)	P (%) of *pfhrp2/3* deletions (%)
Amoah et al. [74]	Africa	38	72.66	27.33	0.49	15.79	NR	NR
Berhane et al. [14]	Africa	31	38	62	NA	100	100	100
Berzosa et al. [21]	Africa	122	84.38	15.62	0.18	75.41	78.69	66.39
Beshir et al. [49]	Africa	7	82.44	17.5	0.15	85.71	14.29	NR
Bharti et al. [24]	Asia	50	76	NA	NA	72.00	54.00	50.00
Funwei et al. [69]	Africa	31	88.53	9.11	0.001	25.81	12.90	12.90
Gupta et al. [22]	Africa	69	85.89	14.11	0.14	1.45	NR	0.0
Koita et al. [28]	Africa	26	95	5.16	0.063	38.46	NR	0.0
Kobayashi et al. [52]	Africa	69	NA	1.7	NA	4.35	0.00	0.0
Kozycki et al. [11]	Africa	140	91.8	NA	NA	22.86	NR	NR
Kumar et al. [50]	Asia	2	95.83	4.17	NA	100	100	100
Maltha et al. [13]	South America	21	71.60	28.38	NA	90.48	0.00	90.48
Nderu et al. [9]	Africa	91	93.8	6	0.08	0	0.00	NR
Owusu et al. [47]	Africa	138	80.78	20.97	0.002	4.35	5.80	4.35
Parr et al. [46]	Africa	783	71.55	28.45	NA	19.03	NR	NR
Pati et al. [25]	Asia	58	84.90	15.1	0.155	48.28	41.38	29.31
Plucinski et al. [51]	Africa	5	81	NA	NA	40.00	NR	20.00
Thomson et al. [12]	Africa	173	89.34	10.66	0.1368	5.20	1.73	1.73
Willie et al. [30]	Africa	8	96	12.9	0.144	0.00	NR	NR
Wurtz et al. [16]	Africa	7	94.07	5.90	NA	42.86	85.71	28.57

*N* sample size, *P* prevalence among RDT with negative results, *NA* not applicable, *NR* not reported

#### Reported prevalence of pfhrp2, pfhrp3 and pfhrp2/3 deletions for false-negative HRP2-based RDT results

**Fig. 13:**
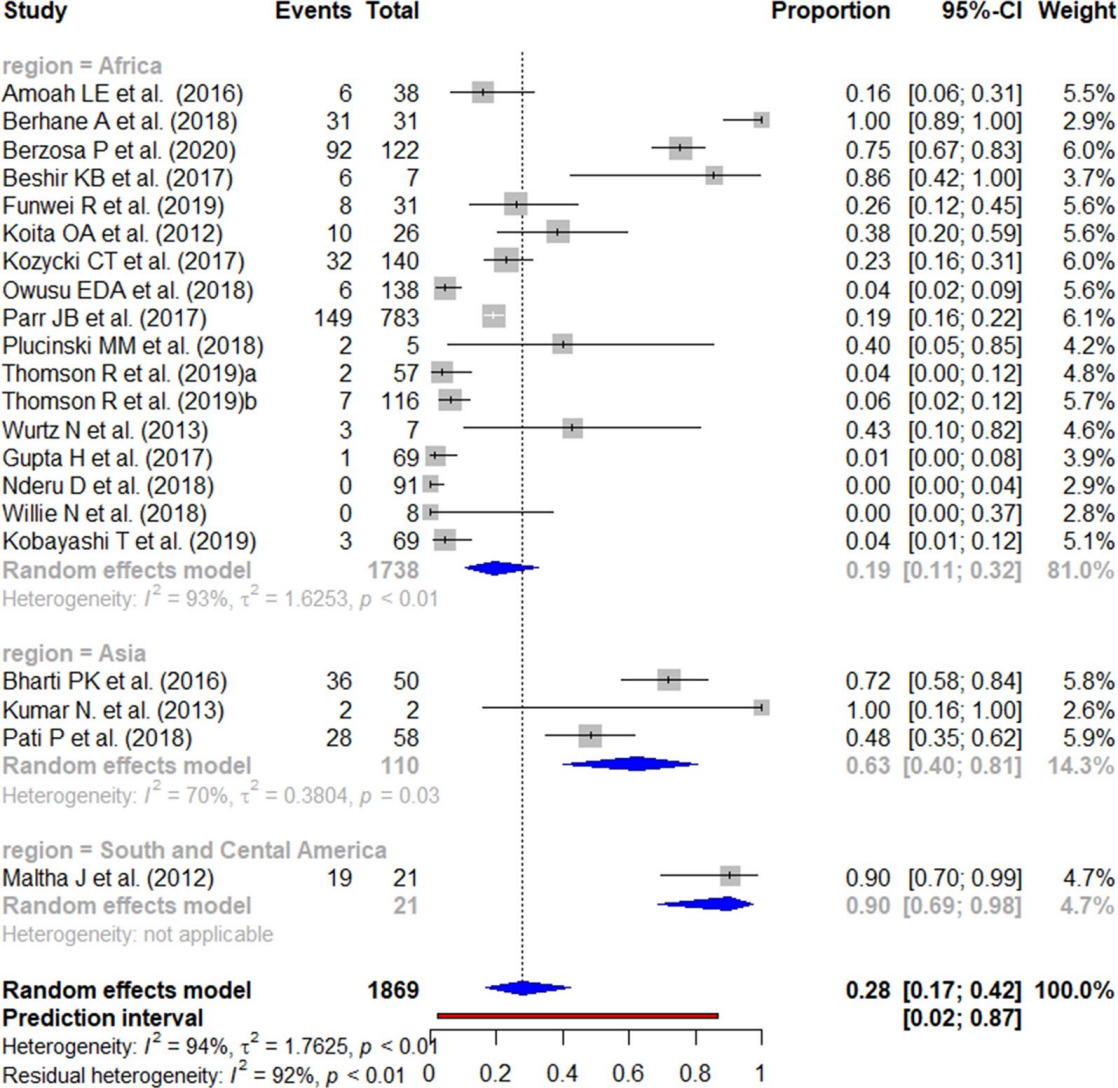
Forest plot showing the prevalence of *pfhrp2* deletion among HRP2-based RDT false negatives worldwide. Each grey square represents one study (size proportional to relative weight) and black lines represent the effect and its confidence interval. Pooled prevalences are represented as blue diamonds for each WHO region

The reported prevalence of *pfhrp2* deletion among false-negative HRP2-based RDT results ranged from 0 to 100% in Africa (Fig. 13). In contrast, in Asia and Central and South America, the lowest published prevalence was 90% and 48%, respectively.

**Fig. 14:**
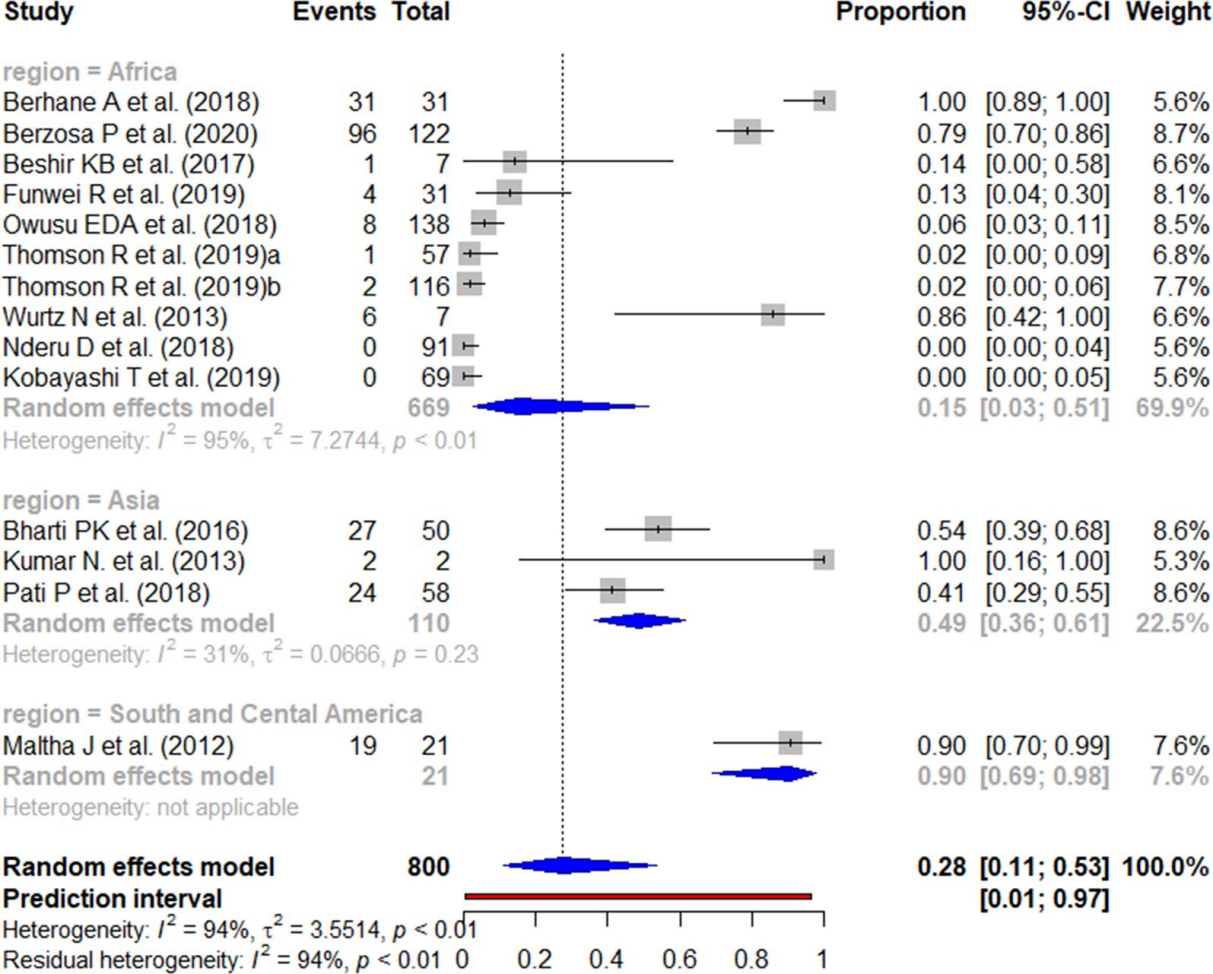
Forest plot showing the prevalence of *pfhrp3* deletion among HRP2-based RDT false negatives worldwide. Each grey square represents one study (size proportional to relative weight) and black lines represent the effect and its confidence interval. Pooled prevalences are represented as blue diamonds for each WHO region

**Fig. 15:**
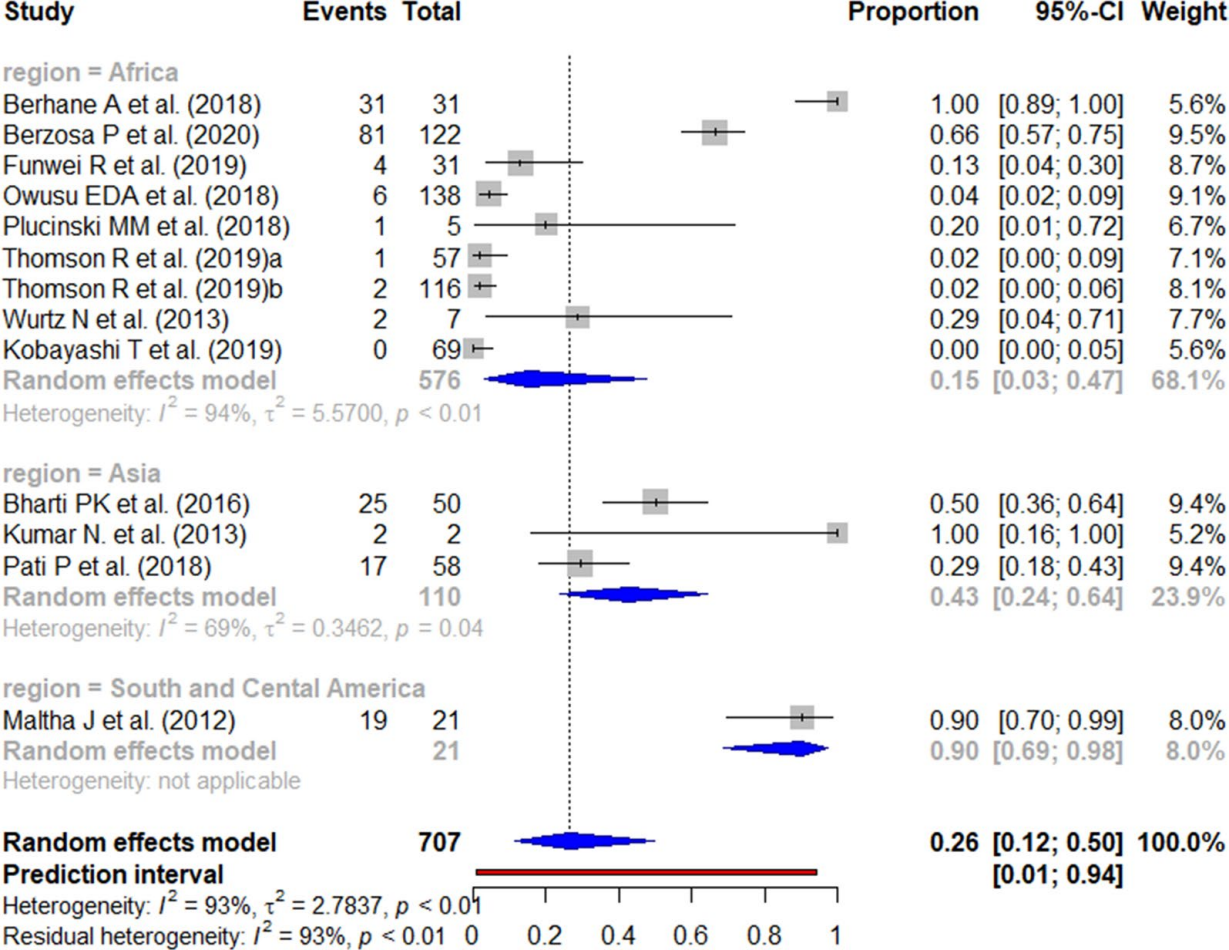
Forest plot showing the prevalence of *pfhrp2* & *pfhrp3* double deletion among HRP2-based RDT false negatives worldwide. Each grey square represents one study (size proportional to relative weight) and black lines represent the effect and its confidence interval. Pooled prevalences are represented as blue diamonds for each WHO region

The published data for the *pfhrp3* deletion (Fig. 14) and *pfhrp2/3* double deletion (Fig. 15) among false-negative RDT results varied widely between studies.

## Discussion

This review describes and assesses the recent literature about the emerging global issue represented by false-negatives related to *pfhrp2*, *pfhrp3* and *pfhrp2/3* deletions in RDT. The study focused on three main concerns. Firstly, the methodology used to study the deletions in *pfhrp2* and *pfhrp3* genes as the standardization of methodologies could increase the reliability and comparability of results. Secondly, the aggregated prevalence of deletion of *pfhrp2*, *pfhrp3* and *pfhrp2/3* genes, with an in-depth analysis for studies conducted in Africa. Thirdly, the implications of *pfhrp2*, *pfhrp3* and *pfhrp2/3* genes in false-negative RDT results. As a consequence, this review provides an original global and complete overview of the current situation of these gene deletions. Two previous systematic reviews had partially targeted *pfhrp2* and *pfhrp3* gene deletions in a specific context (Africa and India) [64, 65], but not worldwide, and a further review was more focused on methodological aspects; however, fewer articles have been included [66].

### Methodologies used to study the deletion of *pfhrp2*, *pfhrp3* and *pfhrp2/3* genes

Despite the WHO protocol for the surveillance of *pfhrp2*, *pfhrp3* and *pfhrp2/3* gene deletion, wide methodological differences were identified between studies [1]. Firstly, studies were conducted in different populations, thus meaning that sociodemographic characteristics might have influenced the prevalence of deletions [66]. For example, it is known that a higher prevalence of suppression has been reported in children [46]. Moreover, although surveillance is recommended only for symptomatic patients, the majority of published studies involved both the asymptomatic and symptomatic population. The WHO recommendation regarding taking samples from febrile patients is based on ensuring good sample quality for the analysis [67]. However, if the deletion involves fitness-cost for the parasites, which has not yet been explored, it is expected that the symptoms will affect the detected deletion prevalence [5].

Another important difference between study populations lies in the malaria transmission season for data collection, which is not provided in all studies. Thus, the prevalence of deletion varies between low and high malaria transmission seasons as malaria is more prevalent during the rainy season [17]. Only one of the articles that reports collection over a whole year stratifies the results by transmission season [25].

Finally, the reliability of results from sample-based studies may also be affected by sample size and geographical location. With regard to sample size, the results varied from too small a sample to provide significant results to a sufficiently large sample to provide significant conclusions, thus limiting the ability to compare statistical significance between studies [64]. Moreover, the varied geographical settings, in terms of location and setting characteristics, make it difficult to assume that studies are representative of the country in which they were conducted, thus also meaning that international comparisons are unsuitable and unreliable.

All studies, except one, included a molecular analysis of deletions (the majority by PCR), with only a few using microsatellites. Although almost all studies used PCR, a wide variety of primers or conditions were used for amplification [64]. Moreover, some articles reported using sequencing, but only for the analysis of sequence variations rather than to confirm gene presence or deletion. This may also have influenced the reliability and comparability of the results.

Another important issue is that some articles only searched for deletions among discordant samples, assuming that most deletions will be found in false-negative RDT results and that it is in this subgroup in which they have an impact. The majority of these studies extrapolated their results to the general sample size [67]. However, this could mean an underestimation of deletion prevalence.

Moreover, for reliable results it is essential to test the quality of DNA. Accordingly, none of the studies that did not test the quality of their samples can assume the reliability of their results [37, 68]. To achieve that, studies should report the amplification of at least two different *P. falciparum* genes, for example *pfmsp1* and *pfmsp2* [12, 64, 69]*.* This is useful to check the quality of the DNA or the concentration to be amplified in order to ensure that, if PCR for *pfhrp2* and *pfhrp3* does not amplify, there is actually a problem with the DNA used rather than a deletion.

Some studies also analysed parasite density by qPCR, which allows the quality and quantity of the DNA to be tested simultaneously [22, 29]. These studies were able to avoid confusing false-negative RDT results caused by low density with those caused by eliminations [64, 70]. However, as this review shows, the majority of studies (n = 33) did not check the parasite density.

The proven cross-reaction of HRP3, the protein encoded by *pfhrp3*, with PfHRP2-based RDT means that the deletions in this gene, and the combination of both deletions (*pfhrp2/3*), must be evaluated to address the performance of RDT [71]. As such, the majority of studies included analysis of the *pfhrp3* gene. Moreover, it is relatively common for studies to also include an analysis of flanking genes for *pfhrp2* and *pfhrp3* [20, 63]. The rapid appearance of deletions in these regions [41], and their possible relation with the multiple origins of the *pfhrp2* deletion, has been described [57].

### Pooled prevalence of *pfhrp2*, *pfhrp3* and *pfhrp2/3* deletions

The WHO recommends not to use HRP2-based RDT as a diagnostic tool when the prevalence of deletions is > 5% given that, in this situation, the efficacy of RDT would be highly compromised [72]. According to the results of the meta-analysis in this study, the prevalence of *pfhrp2*, *pfhrp3* and *pfhrp2/3* deletions is only compromised in South and Central America, whereas other WHO Regions report a significantly lower prevalence. Nevertheless, as the map created by WHO showed, the absence of representative studies in all malaria endemic areas makes difficult to assess global conclusions (https://apps.who.int/malaria/maps/threats/). This is important because more than 90% of malaria cases in Africa are caused by *P. falciparum*, whereas in America the principal aetiological agent is *P. vivax* [1]. As such, the effects caused by a decrease in the efficacy of HRP2-based RDT would potentially be more critical in Africa.

With regard to the high prevalence of deletions in South America, previous studies have shown that these deletions are not equally distributed, with the Amazonas area concentrating the highest prevalence of deletions [20, 59]. Moreover, various studies have reported an absence of mutations in some regions, thus supporting this unequal distribution [19, 73].

Geographical differences were also found between studies conducted in Africa. This could be explained by the malaria transmission season during sample collection [14], as well as by geographical epidemiological differences in terms of baseline prevalence, resistance, public health strategies [38]. In contrast, according to the results showed in this paper, there were no statistically significant differences in pooled prevalence between Africa’s geographical regions, although this might be related to the lack of more representative studies. Thus, it is essential to correctly characterize the status of deletions in each Africa region due to their different characteristics. To assess the actual national prevalence of deletions, it would be advisable to perform national studies with sufficient geographical representation [46, 74] and during specific seasons [17].

It should also be taken into account that, when the genetic diversity is low (either during the low transmission season or in zones with low prevalence of malaria), deletions will be more likely to be selected, thus making it easier for them to spread [60, 75]. Although malaria is endemic in the majority of countries in Africa, it has different characteristics (transmission, treatment strategy) and there are also countries with strong malaria-control programs in place. This could be a potential scenario for the spread of deletions, which would have severe implications and would stop progress in controlling the disease.

The genetic diversity and geographical distribution give information about the selection process for deletions and their spread. The use of RDT for diagnosis for 10 years could drive up the deletion of *pfhrp2* and *pfhrp3* genes [27]. In this regard, one study reported that the continued use of HRP2-based RDT alone will quickly result in the selection of deleted parasites [76]. The same article also reported that this pressure decreases when an HRP2 diagnosis is combined with the detection of another protein. In the field, the majority of RDTs currently detect at least two proteins, mainly pLDH and PfHRP2 [77, 78]. This could explain why, although the majority of countries have significantly increased the use of HRP2-based RDT since 2010, there is no agreement about selection pressure. Thus, some articles support the lack of strong evidence about selection pressure for mutants lacking the *pfhrp2* and *pfhrp3* genes [15, 79], whereas others suggest that the impact of selection pressure favour parasites with deletions [27, 31]. It was also evaluated the possible change in deletions over time but found no relationship between time and deletions, probably because of the lack of a sufficient number of studies to analyse this properly.

The results of the meta-analysis also showed a higher prevalence of deletions among children, as reported previously [17]. This may be because children usually have infections with less genetic diversity, which is especially concerning due to the high mortality in children [80].

Another important issue addressed in this review was the possible differences in deletions between symptomatic and asymptomatic patients. This question was answered by assuming that samples from health centres could be considered to be symptomatic. Previous research suggests the potential fitness costs of the *pfhrp2* deletion, thus meaning a greater prevalence of deletion among the asymptomatic population [27]. This was supported by studies reporting a higher prevalence of deletions among asymptomatic patients [28, 46]. In contrast, one study reported no association between *pfhrp2/3* mutants and fitness cost [7]. The results of the meta-analysis support the first idea, namely that the asymptomatic population carries this deletion more frequently, as found in studies involving the general population. Either way, there is no sufficiently strong epidemiology or laboratory evidence to support this, probably due to the lack of further studies. To address these concerns, further studies analysing patients according to their clinical characteristics are still required.

### Impact of pfhrp2, pfhrp3 and pfhrp2/3 deletions on false-negative RDT results

The major threat of deletions in *pfhrp2* and *pfhrp3* genes is their effects on the efficacy of RDT. As a result of following the test-based treatment strategy, patients who are mistakenly diagnosed will not receive the correct treatment on time, which is an essential condition for successful recovery and, therefore, for improving malaria control in affected areas [16, 22]. In this regard, it has been suggested that a strong test-based treatment strategy favours the selection of *pfhrp2* and *pfhrp3* mutants [46]. This hypothesis, which is based on escape from treatment due to a lack of diagnosis, was supported by a previously published simulation model [76].

The majority of studies have reported a relationship between gene deletions and false-negative RDT results by analysing the deletions only among false-negative RDT samples. This approach is useful for that aim but may not be suitable for characterising the deletion status as RDT could be positive, even if *pfhrp2* is deleted, due to a cross-reaction with *pfhrp3*. In addition, a higher prevalence of deletions is expected in these samples [11]. As such, these studies might have underestimated *pfhrp2* and *pfhrp3* single deletions, the prevalence of which could be important for predicting the future threat to RDT efficacy.

The impact of deletions on HRP2-based RDT results could be related to the lower sensitivity and higher false-negative rate of RDT. The fact that parasites with a *pfhrp2/3* deletion are not detected means that the sensitivity of RDT decreases [5]. Although a relationship between RDT and gene deletions has been reported in this review, it have not been possible to determine a directly proportional relationship, probably as a consequence of the high inter-study heterogeneity.

## Limitations and strengths of the study

The principal limitation of this review was the high inter-study heterogeneity. Moreover, some articles used a subset of their samples, usually the subgroup of false-negative RDT results, to detect the deletion and calculate the prevalence of deletion in the whole sample using only this value. This could lead to an underestimation of the prevalence. To decrease this heterogeneity, it was performed a subgroup analysis by health facility or general population, which could introduce a bias. Moreover, the wide variety between studies and the way in which their results are reported has made it difficult to interpret and to standardize the results for systematic review and meta-analysis. Finally, although all the obtained results from the publication bias assessment were not significant, some bias related to the absence of complete data or the need to report significant results may nevertheless be present.

In summary, this review represents a complex and global the first approach to characterize the worldwide status of these deletions taking into account different epidemiological variables combined with the main methodological aspects of the different study designs. As such, it could be used as a reference for future studies.

## Conclusion

This review highlights the concerning prevalence of *pfhrp2*, *pfhrp3* and *pfhrp2/3* gene deletions and their variation worldwide. Although these results are still too limited to reconsider the efficacy of RDT, these deletions represent a serious threat to malaria control as a lack of safe and quality diagnosis means a lack of adequate treatment, thus meaning that uncontrolled infection may increase, along with the morbidity and mortality rates. The impact of these deletions is more severe in regions with a low prevalence of malaria, particularly in those countries close to malaria control or eradication, where regular and systematic surveillance of these deletions as part of national guidelines for malaria control is highly recommended. To combat this threat, this study justifies the need for an RDT that combines two proteins or the combination of HRP2-based RDT with another diagnostic method due to the questionable efficacy of the former. Additionally, it is also highly recommended to combine these strategies with evaluations of diagnostic quality.

This review has also highlighted the need to better characterize the threat posed by these deletions. In this regard, a standardized methodology for all studies could play a key role in increasing the understanding of these deletions, their transmission dynamics and their modifiers and associated effects, thereby allowing a comparison between regions in terms of epidemiological variables. Moreover, more genetic studies could help to better characterize these genes and their dynamics.

It is also important to recommend that the gold standard diagnosis in endemic countries, namely microscopy, should not be neglected. Microscopy has been side-lined in many places due to the introduction of RDTs. However, this diagnostic method is still cheap and gives a malaria diagnosis in less than 30 min. As such, the availability of high quality rapid tests together with quality microscopy would enable diagnostic and reference centres to offer better health care to the population.

## Supplementary Information

**Additional file 1: Table S1.** The PRISMA checklist.

**Additional file 2: Table S2.** Pooled prevalence of *pfhrp2*, *pfhrp3* and *pfhrp2/3* deletions among all *P. falciparum* cases by WHO region, population symptomatology, age of population and collection season.

**Additional file 3: Figure S1.** Contour-enhanced funnel plot of studies included in the *pfhrp2* deletion meta analysis.

**Additional file 4: Figure S2.** Contour-enhanced funnel plot of studies included in the *pfhrp3* deletion meta analysis.

**Additional file 5: Figure S3.** Contour-enhanced funnel plot of studies included in the *pfhrp2* &* pfhrp2* double deletion meta analysis.

**Additional file 6: Figure S4.** Assessment of inter-study heterogeneity for the *pfhrp2* deletion analysis.

**Additional file 7: Figure S5.** Assessment of inter-study heterogeneity for the *pfhrp3* deletion analysis.

**Additional file 8: Figure S6**. Assessment of inter-study heterogeneity for the *pfhrp2* & *pfhrp3* double deletion analysis.

## Data Availability

All data generated during this study are included in this published article and its Additional files. The datasets used and analysed (extraction table) during the current study are available from the corresponding author on reasonable request.

## Abbreviations

*pfhrp2*: *Plasmodium falciparum* Histidine-rich protein 2
*pfhrp3*: *Plasmodium falciparum* Histidine-rich protein 3
WHO: World Health Organization
RDT: Malaria rapid diagnostic test
HRP2: Histidine-rich protein 2
HRP3: Histidine-rich protein 3
