# Supplementary material for: Impact of Plasmodium falciparum pfhrp2 and pfhrp3 gene deletions on malaria control worldwide: a systematic review and meta-analysis
Source: Malar J. 2021 Jun 22;20:276. doi: 10.1186/s12936-021-03812-0 (PMC8220794; doi:10.1186/s12936-021-03812-0)
Supplement: Supplementary file 2 — Additional file 2: Table S2. Pooled prevalence of pfhrp2, pfhrp3 and pfhrp2/3 deletions among all P. falciparum cases by WHO region, population symptomatology, age of population and collection season. [file 12936_2021_3812_MOESM2_ESM.pdf]

Table S2. Pooled prevalence of *pfhrp2*, *pfhrp3* and *pfhrp2/3* deletions among all *P. falciparum* cases by WHO region, population symptomatology, age of population and collection season.

	N	***pfhrp2* deletions**		I^2 (%)	N	***pfhrp3* deletions**		I^2 (%)	N	***pfhrp2/3* deletions**		I^2 (%)
		P (%)	95% CI			P (%)	95% CI			P (%)	95% CI	
**WHO region**												
Africa	18	3.77	2.61 – 5.41	80.9	15	4.22	1.78 – 9.68	96.7	11	3.63	2.03 – 6.41	84.0
Central and South America	9	13.19	8.42 – 20.09	83.7	7	37.05	27.72 – 47.47	88.4	6	5.66	2.87 – 10.87	75.0
Asia	6	4.82	2.28 – 9.91	89.0	3	1.67	0.18 – 13.85	92.6	5	2.20	0.97 - 4.94	79.9
Cochran's Q (df)	18.10 (2)				33.40 (2)				3.09 (2)			
p-value	**0.0001**				< **0.0001**				0.2135			
**Symptomatology of sample population**												
Symptomatic	12	8.42	5,26 – 13.19	82.4	10	19.30	9.12 – 36.32	97.4	6	4.61	2.09 – 9.86	89.8
Asymptomatic	1	9,88	5.02 – 13.19	NA	2	6.03	0.10 – 79.69	88.6	2	3.94	0.19 – 46.57	79.8
General (Mixed)	20	4.35	2.90 – 6.47	90.4	13	4.24	1.72 – 10.07	82.4	14	2.68	1.55 – 4.58	79.6
Cochran's Q (df)	6.55					6.83 (2)				1.27 (2)		
p-value	0.0379					0.0329				0.5312		
**Age of sample population**												
Children	6	8.31	5.11 – 13.22	77.2	7	29.45	17.30 – 45.45	93.1	6	7.41	4.26 – 12.58	64.6
General Population	27	4.56	3.05 – 6.77	90.6	18	4.57	2.11 – 9.63	97.3	16	2.77	1.69 – 4.52	85.5
Cochran's Q (df)	3.54				16.12 (1)				6.80 (1)			
p-value	0.0600				< **0.0001**				0.0091			
**Collection season**												
High transmission	10	4.34	2.35 – 7.87	88.0	7	4.68	1.05 – 18.58	93.5	8	1.48	0.78 – 2.77	65.2
Low transmission	1	5.34	4.37 – 6.50	NA	2	13.30	2.11 – 52.26	99.3	2	7.80	2.81 – 19.83	96.3
Both or annual transmission	22	5.69	3.67 – 8.71	90.3	16	12.52	7.46 – 20.27	90.3	16	5.63	3.51 – 8.91	69.0
Cochran's Q (df)	0.53				1.67 (2)					13.34 (2)		
p-value	0.7679				0.4337					**0.0013**		

N: Number of articles included; P: Prevalence (%), 95% CI: 95% Confidence Interval; I^2: I – square, index of heterogeneity; *Cochran's Q* : Indicates the heterogeneity between subgroups.
