# Supplementary figures and images for: Impact of Plasmodium falciparum pfhrp2 and pfhrp3 gene deletions on malaria control worldwide: a systematic review and meta-analysis
Source: Malar J. 2021 Jun 22;20:276. doi: 10.1186/s12936-021-03812-0 (PMC8220794; doi:10.1186/s12936-021-03812-0)

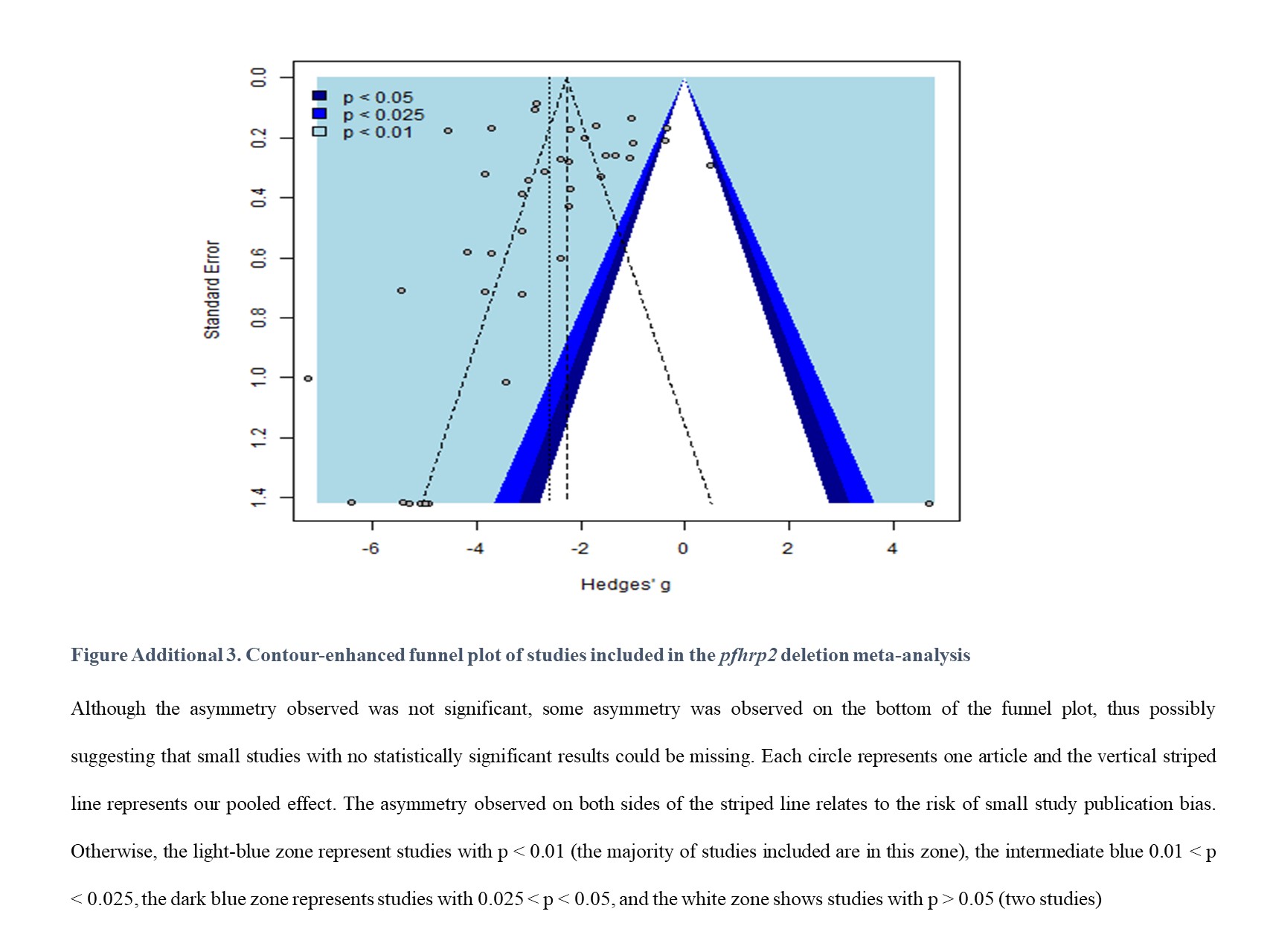

Supplement: Supplementary file 3 — Additional file 3: Figure S1. Contour-enhanced funnel plot of studies included in the pfhrp2 deletion meta analysis. [file 12936_2021_3812_MOESM3_ESM.jpg]

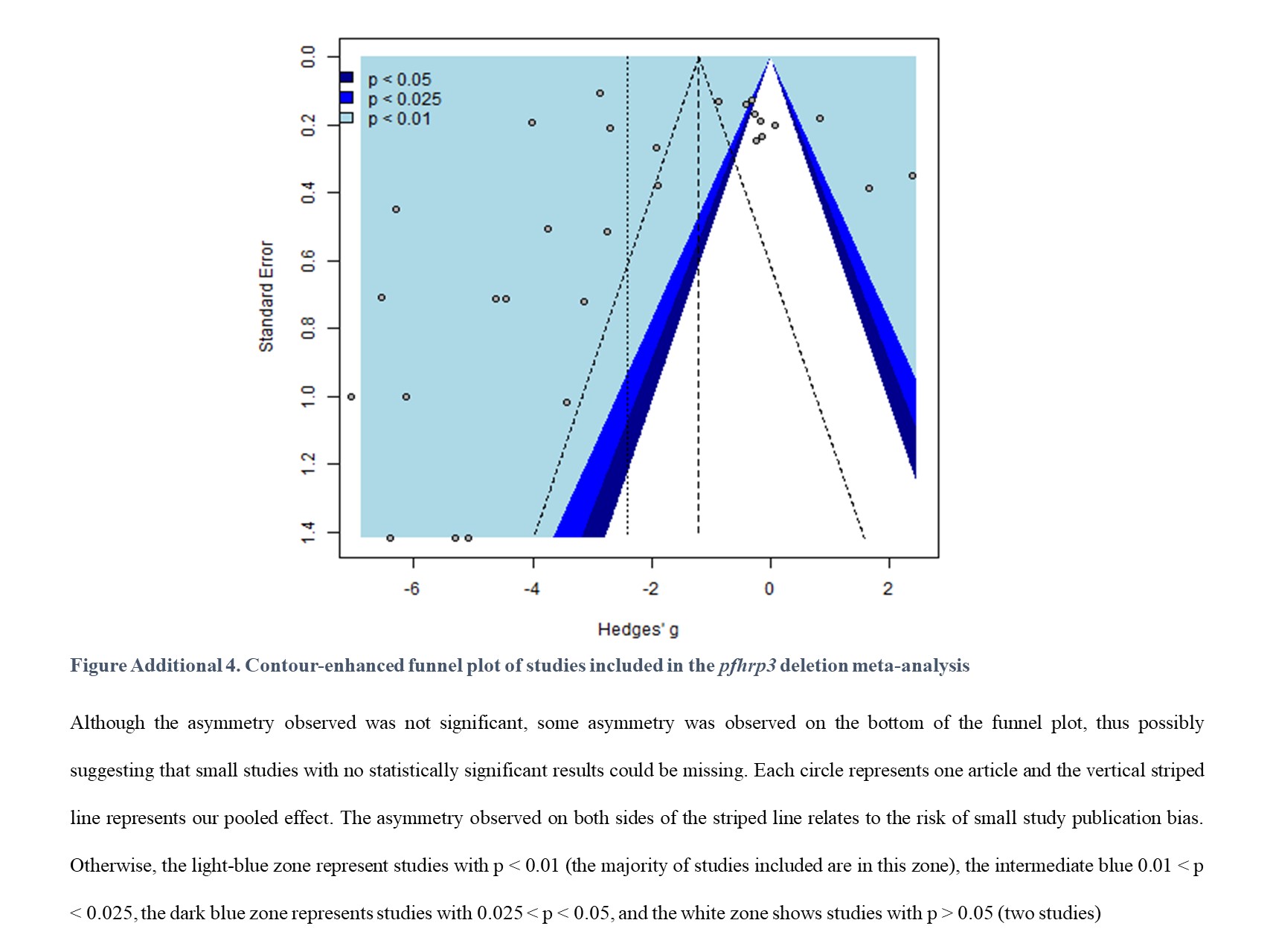

Supplement: Supplementary file 4 — Additional file 4: Figure S2. Contour-enhanced funnel plot of studies included in the pfhrp3 deletion meta analysis. [file 12936_2021_3812_MOESM4_ESM.jpg]

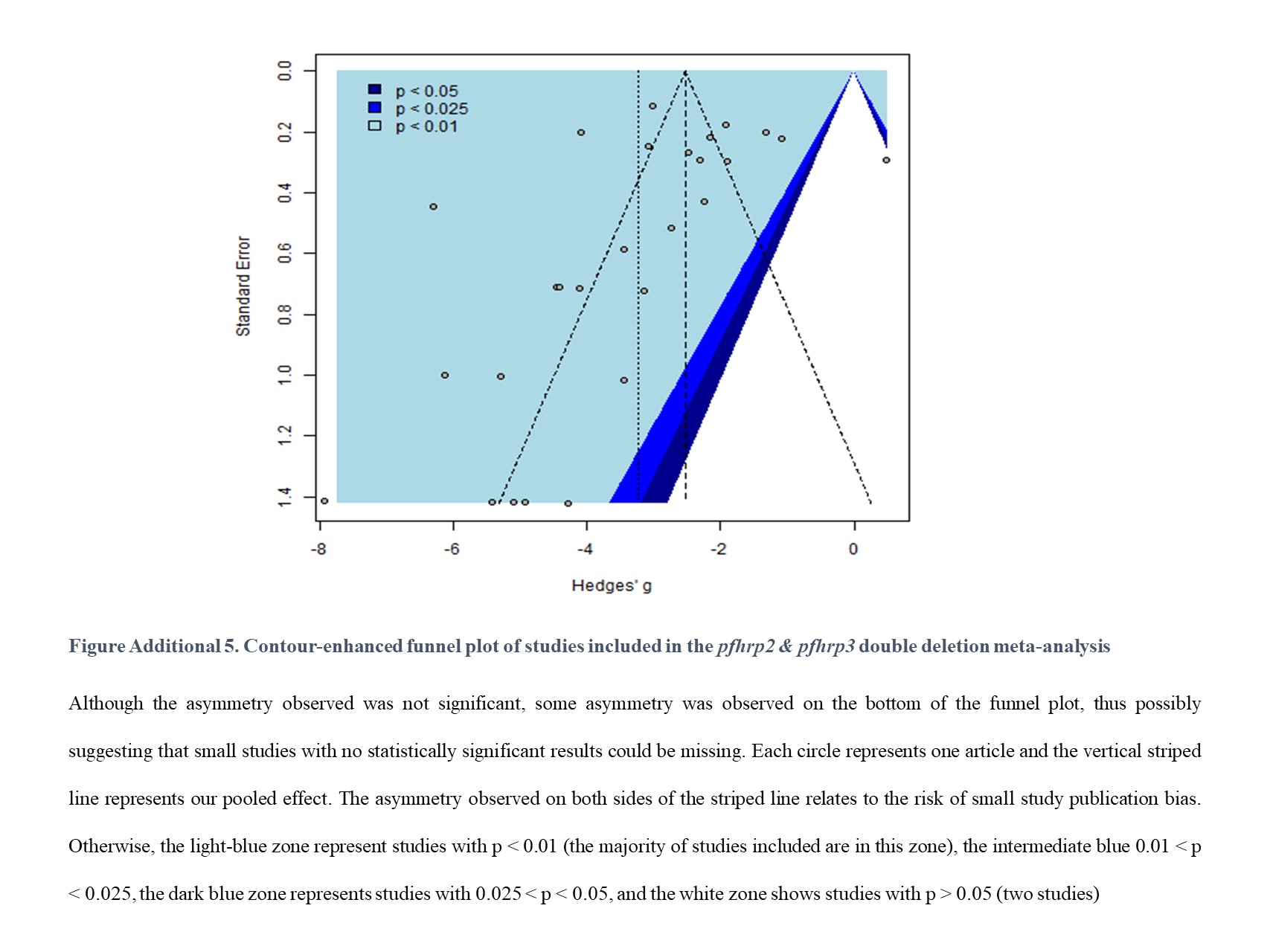

Supplement: Supplementary file 5 — Additional file 5: Figure S3. Contour-enhanced funnel plot of studies included in the pfhrp2 & pfhrp2 double deletion meta analysis. [file 12936_2021_3812_MOESM5_ESM.jpg]
