# Supplementary material for: Impact of Plasmodium falciparum pfhrp2 and pfhrp3 gene deletions on malaria control worldwide: a systematic review and meta-analysis
Source: Malar J. 2021 Jun 22;20:276. doi: 10.1186/s12936-021-03812-0 (PMC8220794; doi:10.1186/s12936-021-03812-0)
Supplement: Supplementary file 6 — Additional file 6: Figure S4. Assessment of inter-study heterogeneity for the pfhrp2 deletion analysis. [file 12936_2021_3812_MOESM6_ESM.pdf]

Figure S4. Assessment of inter-study heterogeneity for the *pfhrp2* deletion analysis. The combination of both analyses resulted in the exclusion of the following articles: Berhane A et al. (2018), among studies from health facilities, and Gupta H et al. (2017), among studies in the general population.

Fig. S4a, S4b Baujat plot for studies included in the meta-analysis. The horizontal axis indicates the contribution of each study to the overall heterogeneity (measured by Cochran's Q), therefore studies on the right-hand side increase the heterogeneity more. The vertical axis indicates the influence of each study on the pooled proportion. Studies at the top have a greater influence on the pooled result.

Fig. S4a. Baujat plot for studies included in the meta-analysis of *pfhrp2* deletions among samples from health facilities.

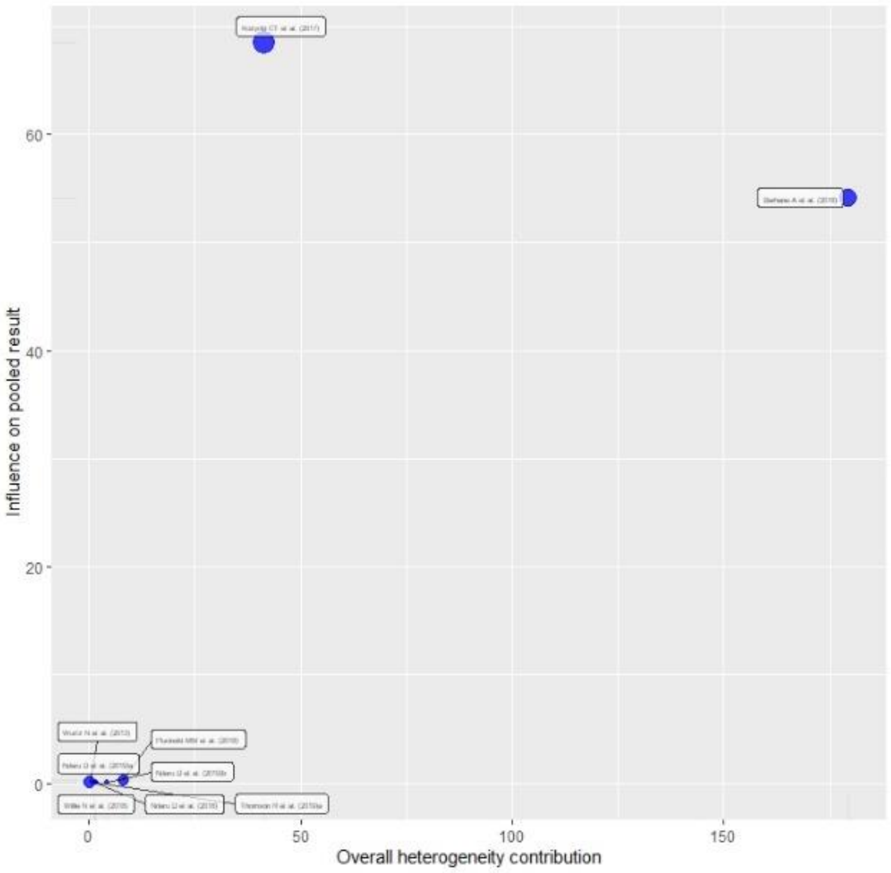

Fig. S4b. Baujat plot for studies included in the meta-analysis of *pfhrp2* deletions among samples from the general population.

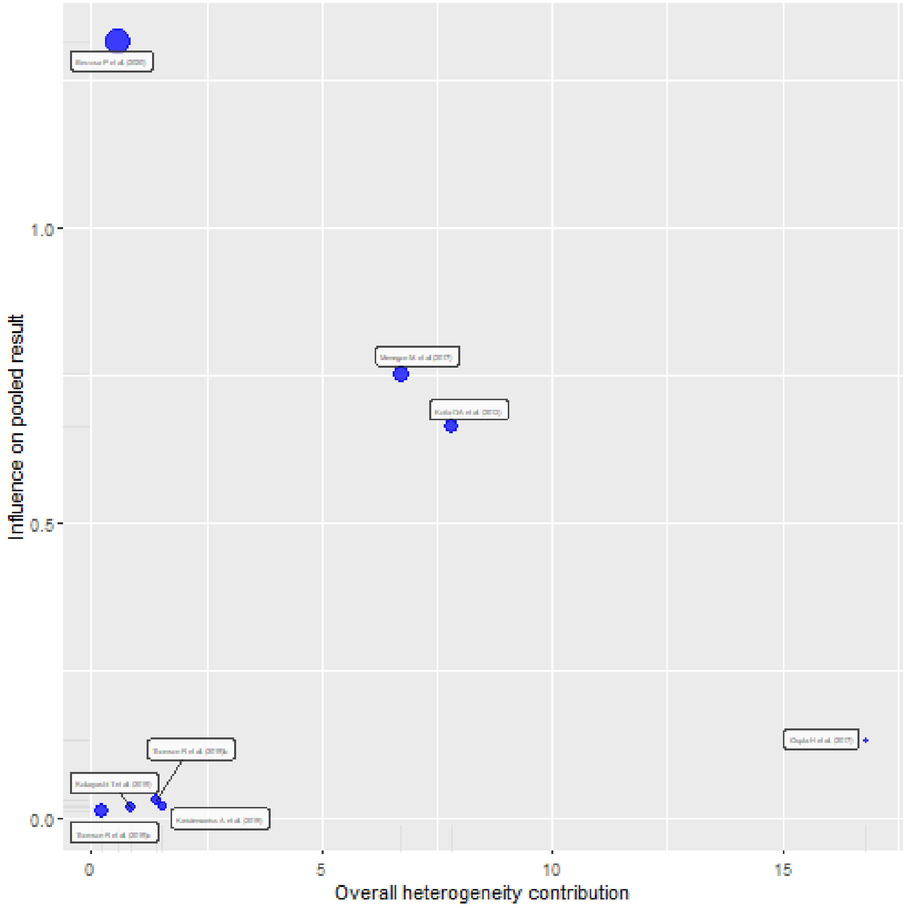

Fig. S4c, S4d. Forest plot for *pfhrp2* deletion prevalence according to the outliers analysis. All results whose confidence interval did not overlap with the prediction interval were excluded. These articles had a relative weight of 0%.

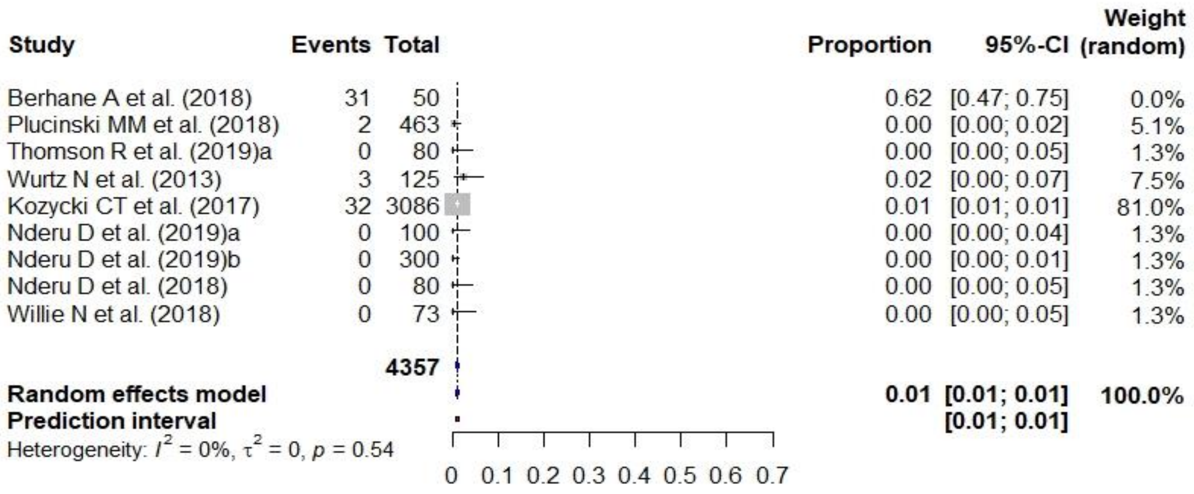

Fig. S4c. Forest plot for studies included in the meta-analysis of the *pfhrp2* deletion in samples from health facilities, with outliers identified.

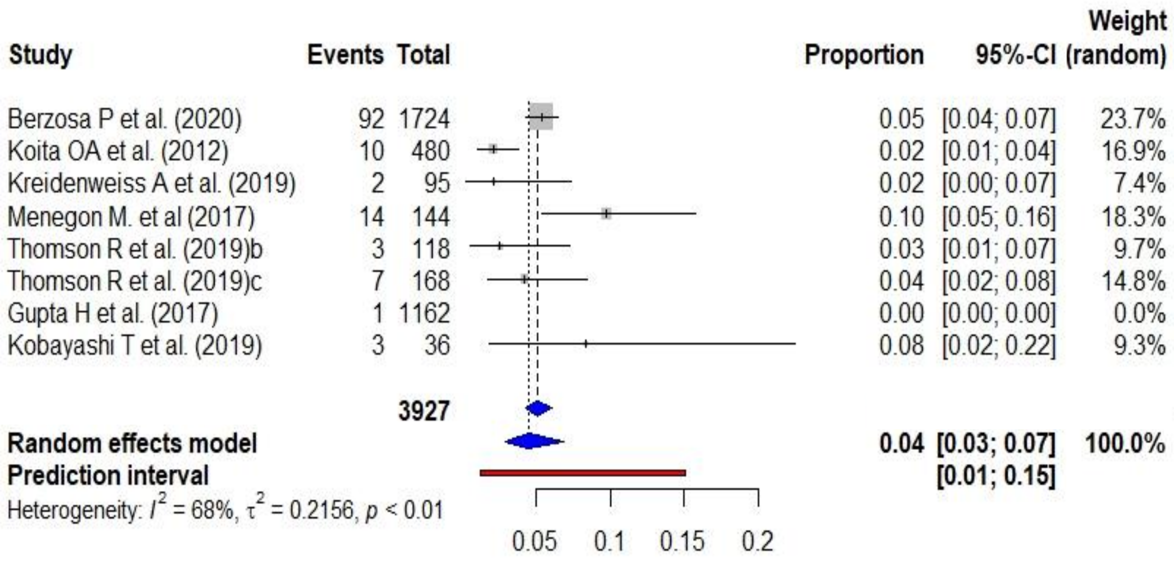

Fig. S4d. Forest plot for studies included in the meta-analysis of the *pfhrp2* deletion in samples from the general population, with outliers identified.
